# Neck Motion and Injuries of Small Females and Midsize Males in Frontal Impacts at Two Severities

**DOI:** 10.1007/s10439-026-03981-6

**Published:** 2026-01-30

**Authors:** Corina Espelien, John-Paul Donlon, Jeesoo Shin, Mary Gallaher, Sara Sochor, Junior Noss, Randolff Carpenter, Pablo Gracia Cemborain, Jason Forman

**Affiliations:** Center for Applied Biomechanics, Department of Mechanical and Aerospace Engineering, University of Virginia, Charlottesville, VA USA

**Keywords:** Biomechanics, Spine, Neck, Cadaver, Female, Frontal impact

## Abstract

**Purpose:**

Head and neck injuries remain a critical area of concern in automotive safety. To inform injury prediction tools, like crash test dummies, neck validation data are needed. While neck response information for midsize males is available, anthropometry-specific data are needed for other body shapes, including small females. The objective of this study is to characterize the neck response (head and neck kinematics and neck injuries) for small females and compare it to the response of midsize males in the same testing conditions.

**Methods:**

Six small adult female post-mortem human subjects (PMHS) and three midsize adult male PMHS were each tested twice, once at 3-g (20 km/h) and again at 8-g (43 km/h) in frontal impacts. Motion-capture measurements of the skull and first thoracic vertebrae (T1) were analyzed to calculate head and neck motion.

**Results:**

Females had less downward and less forward head excursion than males for both 3-g and 8-g impact tests. The decreases in peak head responses between the 8-g and 3-g tests were larger for small females than for males, with the largest decrease of 30% for small female downward excursion. Rotation responses were similar for females and males at 3-g. At 8-g; the overall range of T1 pitch was similar for both sexes, although males exhibited an initial rearward T1 pitch. No injuries were found for the male PMHS, while two of the female PMHS had cervical spine injuries.

**Conclusion:**

These kinematic and injury data can be used for injury prediction tool neck validation.

**Supplementary Information:**

The online version contains supplementary material available at 10.1007/s10439-026-03981-6.

## Introduction

Human surrogates, like anthropometric test devices (ATDs) and computational human body models (HBMs), are used in regulatory and consumer testing to assess car safety by predicting a biofidelic (or human like) response. These surrogates can represent a variety of anthropometries, or body sex and sizes. Two of the common target anthropometries are the midsize (50^th^ percentile) male [1, 2] and small (5^th^ percentile) female [3–5]. ATDs and HBMs need to have biofidelic heads and necks so that vehicle safety systems, like airbags, are built around a representative human response. Neck biomechanics dictate head motion, which influences contact with interior vehicle structures and contact or inertial head injury. Head injuries, including traumatic brain injuries (TBIs), are common in motor-vehicle collisions (MVCs) [6]. MVCs are the second leading cause of non-fatal TBIs, accounting for 17% [7]. Additionally, the outcomes following TBI can be severe; there are over 16,000 fatal TBIs related to MVCs every year in the USA, the majority of which were male fatalities [8]. Controlling for other crash and occupant factors, females are 56% less likely than their male counterparts to suffer a severe TBI, while females are 76% more likely than their male counterparts to suffer a moderate TBI [9].

Additionally, the inertia of the head (a cantilevered mass for a restraint vehicle occupant) can load the neck and cause injury. Literature on neck injuries often focuses on sequalae of low-severity rear impacts[10]; however, around one in three MVC-related neck injuries are from frontal impacts [11]. Thus, neck injuries from frontal impacts remain a safety concern. Compared to older car model years, more recent model years show a significantly decreased odds ratio of cervical spine injury (OR = 0.70) [9]. This indicates that modern safety systems (in this case, for car model years from 2009 onwards) can be designed and optimized to protect the neck. However, these safety gains may not be distributed across the population evenly; the same Forman et al. study found that females are 99% more likely to sustain a cervical spine injury compared to males [9].

The neck is compromised of the cervical spine (the seven most superior vertebrae) and surrounding soft tissue. Males and females exhibit anatomical differences in their necks. External measures, like neck circumferences, as well as image-based measures, like vertebral widths, are larger for size-matched males compared to females [12, 13]. Males also have larger volumes of soft tissue in the neck, both in general measures (i.e., including connective tissue, vasculature, etc.) [14] and in muscle-specific measures [15]. Males exhibit greater measures of neck muscle force compared to females [16–18], with larger magnitude differences in flexion than extension [19, 20]. Given these sex-based differences in anatomy and physiology, female data are needed to inform female ATDs and HBMs that are used to assess frontal impacts.

The amount of muscle in the neck, including large-volume extensor muscles like the trapezius, may also mean that muscle tensing could influence the head and neck response during an impact. Neck muscle activation times can range from 27 to 84 ms [21–23], which falls within the pulse duration of 100 ms of generic crash pulse curves [24]. For the muscle-dominated neck, the effect of muscle contraction needs to be considered when assessing ATDs and HBMs, given the overlap in muscle activation and crash pulse duration.

Currently, most experimental data that are used in characterizing the neck response under inertial loading is based on male subjects. Specifically, the Naval Biodynamics Laboratory (NBDL) testing has been used as a cornerstone of neck biomechanics. The NBDL dataset describes volunteer impact experiments covering a range of severities up to 15-g impacts (delta-V 60 km/h), making it extremely valuable in capturing the living human response. In the NBDL data, head and T1 kinematics were captured via motion-tracking and surface-mounted accelerometers.

The T1 data from NBDL defines the motion at the base of the neck and can been used as an input for subsystem testing (e.g., head and neck components only) of ATD and HBMs. However, instrumentation errors from the original data due to the relative motion of the surface mounted instrumentation have been described, with manual curve corrections applied by Thunnissen et al. for the T1 rotation [25]. For physical surrogates like ATDs, component testing has typically involved applying an X-direction linear acceleration on a mini-sled. For the THOR-50 M, the sled pulse (peak around 15-g), rather than the T1 pulse, was used as a simplified input condition [1]; for the THOR-05F, the T1 X-direction acceleration (peak around 30-g) from the midsize male NBDL volunteers was used [26].

HBMs more easily allow for the application of additional degrees of freedom for boundary condition inputs compared to physical testing set-ups with ATDs. The Global Human Body Modeling Consortium (GHBMC) M50 and F05 head and neck subsystem models have used NBDL-based linear velocities in X and Z, as well as rotation in Y, as boundary condition inputs for HBM neck validation across multiple impact severities (2-g to 15-g sled accelerations) [27, 28]. For component-level validation, T1 data helps isolate the head and neck response of the surrogate and removes the dependence on the biofidelity of the whole body of the surrogate. Testing surrogates with NBDL T1 inputs allows for comparisons to the original volunteer head kinematics. However, the NBDL T1 data are noisy and have required correction [25] and the original test series does not provide female T1 data. Additionally, applications of the NBDL data to small female surrogate validation have relied on scaling NBDL outcomes [5].

NBDL-level testing (e.g., up to 15-g) is not repeatable with volunteers under modern testing protocols, meaning an analogous set of data with small female volunteers is not possible. However, recent volunteer testing to examine the head and neck response has shed valuable insights. In volunteer studies examining head and neck motion in impact scenarios with and without muscle activation (e.g., relaxed vs. contracted), the contracted condition decreased the resulting head/neck motion compared to the relaxed condition. Male volunteers exhibited less head and neck motion than female volunteers, which is partially attributable to their greater neck muscle strength [29, 30].

Wismans et al., in 1987, aimed to replicate and expand on the NBDL dataset by testing PMHS in a similar configuration [31]. Of the twelve tests performed at the University of Heidelberg, there were data losses for seven tests. Of the remaining five tests, two (1 male, 1 female) were performed at sled accelerations around 15-g, similar to the maximum NBDL volunteer tests, and three (2 male and 1 female) were performed at sled accelerations around 23-g. For the matched condition at 15-g, the resultant T1 acceleration was smaller in magnitude for the PMHS compared to the NBDL volunteers, while the sagittal excursion envelope and head rotation were larger in magnitude for the PMHS compared to the NBDL volunteers. Cervical spine injuries were reported for all five subjects. The Wismans data includes some limitations as an extension of the NBDL condition with PMHS. The anthropometry of the subjects did not seem focus around a specific target; the height and weight for the three male subjects was similar to a midsize male anthropometry, but the height and weight for the two female subjects were larger than small female anthropometry targets. Formaldehyde was injected into the PMHS neck muscles to simulate muscle tone. Lastly, there is a paucity of information on the head and neck positioning and post-test data processing for these tests. The Wismans et al. 1987 data provide some insight into the comparison of PMHS and volunteer kinematics and injuries broadly, but are limited in their application to small female surrogate assessment because of larger height and weight of the female PMHS subjects (compared to a small female target), the effect of injecting formaldehyde [32], and small sample size (n = 1 at 15-g and n = 1 at 23-g).

Mertz & Patrick tested a volunteer and five PMHS in a simulated frontal impact [33]. The sex and anthropometry of the PMHS from these tests were not reported, although the text of the paper discusses the 50^th^ percentile male. These tests focused on the kinetic response of the neck (the moment-angle response at the occipital condyles (OC), shear, and tension forces), including sensitivities to changes in head-borne mass. Each subject (both the volunteer and PMHS) was tested dozens of times, with maximum peak sled accelerations of 9.6-g and 14.0-g for the volunteer and PMHS, respectively. The volunteer response exhibited more hysteresis than the PMHS in the moment-angle response, with a narrowing of the hysteresis at higher impact severity for the volunteer; this was suggested as a convergence of volunteer and PMHS response as high sled accelerations were reached [33]. The peak head rotation of the volunteer was within the range of peak head rotations exhibited by the PMHS. This work quantified a hysteresis difference for lower impact severities between the PMHS and volunteers, highlighting how muscle effects can be quantified as losses in angular work and are an important consideration in neck biomechanics research. However, the focus of Mertz & Patrick was 50^th^ percentile males, thus limiting its use for small female surrogate validation.

More recent work focusing on pediatric kinematics and scaling methods explored comparisons of adult volunteer and PMHS spine motion in a matched low-speed condition of around 9 km/h [34]. The volunteer subjects were all male; the PMHS cohort involved two males and one female. The testing set up and posture described in Lopez-Valdes et al. was more akin to an automotive environment than the aforementioned NBDL, Wismans, and Mertz & Patrick studies. Namely, the posture was slightly reclined and used a 3-point seatbelt with retractor (in contrast to the earlier studies with an upright torso posture and 5-point harness). Compared to the adult volunteers, the adult PMHS exhibited larger magnitude sagittal excursions, including more vertical (upwards) motion of the head, spine, and pelvis compared to the volunteers.

Beeman et al. [35, 36] compared male (approximately 50^th^ percentile) PMHS and volunteers in matched conditions at both 2.5-g and 5.0-g sled accelerations in a vehicle-like environment; unlike Lopez-Valdes et al., Beeman et al. also included a steering wheel in the testing set up. C7 peak excursions for relaxed volunteers and PMHS were greater than tensed volunteers, as were head peak excursions. Both the PMHS and relaxed volunteer excursions exhibited an initial motion that was predominately horizontal (forward), while the tensed volunteers showed combined horizontal and vertical motion. The kinematic differences between PMHS and volunteers from both Lopez-Valdes et al. and Beeman et al. are likely exacerbated by the testing condition (low severity) and restraint environment (3-point seatbelt allowing for increased torso motion compared to NBDL, Wismans, and Mertz & Patrick). Neither study focused on nor included small female anthropometries in their comparisons.

There is a demonstrated need to include female data in head and neck validation sets, due to differences in injury rates, as well as anatomical and physiological variation. Testing female volunteers in NBDL analogous settings is not possible in the modern era; however, female PMHS can capture data that are anthropometric-specific (e.g., accounts for geometry and sex). Building upon the NBDL dataset, which only included male volunteers but tested to high impact levels, can facilitate a deeper understanding of the contributions of muscle tensing to the head and neck response.

In this study, we reproduced the NBDL test condition with PMHS of two anthropometric categories, midsize males and small females. By replicating the boundary conditions of the volunteer NBDL data, the new PMHS data can complement the previously collected volunteer data; through comparison of volunteers and PMHS, additional insight into the effects of muscle tone on neck response is possible. Sled acceleration, restraint environment, and target posture were designed to match selected NBDL conditions. Midsize male PMHS were selected as one target anthropometry to mirror the anthropometry of the original NBDL testing. Small female PMHS were selected as another target anthropometry due to the applicability to advanced biomechanical models, such as computational models like GHBMC-F05 [3, 4] and physical models like the THOR-05F dummy [5, 26, 37]. Here, we present head and neck kinematics and injuries for those small female and midsize male PMHS at two impact severities in a recreation of the frontal NBDL sled configuration. The primary focus of the analysis is to compare kinematics and injuries between small female and midsize male PMHS in matched conditions.

## Materials and Methods

18 frontal impact sled tests were performed (Table 1) on nine PMHS (six small female and three midsize male, Table 2). Each PMHS was tested twice, first in a 3-g, 20 km/h impact and then in an 8-g, 43 km/h impact. Reports and data from these tests are available on the National Highway Traffic Safety Administration (NHTSA) Biomechanics Database, Test Numbers 15212-15229 [38].

**Table 1:** Test matrix with PMHS IDs and sled test numbers for the small females and midsize male PMHS.

	UVA subject ID	UVA test number		NHTSA database test number	
		3-g	8-g	3-g	8-g
Small female	1046F	S0785	S0786	15212	15213
	1042F	S0787	S0788	15214	15215
	1048F	S0789	S0790	15216	15217
	1051F	S0797	S0798	15224	15225
	1054F	S0799	S0800	15226	15227
	1049F	S0801	S0802	15228	15229
Midsize male	1032M	S0791	S0792	15218	15219
	1038M	S0793	S0794	15220	15221
	1031M	S0795	S0796	15222	15223

**Table 2:** PMHS information.

	UVA subject ID	Stature [mm]	Mass [kg]	Age [yr]	Cause of death
Small female	1046F	1640	57	69	Glioblastoma
	1042F	1620	50	75	Amyotrophic Lateral Sclerosis (ALS)
	1048F	1660	42	71	Ruptured abdominal aortic aneurysm
	1051F	1690	45	73	Pancreatic cancer
	1054F	1595	41	63	End stage renal disease
	1049F	1540	55	69	Hypoxic ischemic leukoencephalopathy
	Mean	1624	48	70	–
	St. dev.	53	7	4	–
	Target	1470-1620	39-54	≤ 67	–
Midsize male	1032M	1770	74	72	Alzheimer’s
	1038M	1810	71	62	Acute Myeloid Leukemia
	1031M	1750	77	76	Metastatic prostate cancer
	Mean	1777	74	70	–
	St. dev.	31	3	7	–
	Target	1690-1820	67-84	≤ 67	–

PMHS acquisition and handling procedures were approved by the University of Virginia Institutional Review Board for Human Surrogate Use (IRB-HSU) Committee. PMHS were frozen until testing, screened for blood-borne pathogens, and handled using universal precautions [39]. Full body computed tomography (CT) scans of the PMHS were used to verify the absence of gross abnormalities and pre-existing injuries in the neck before testing.

**Fig. 1:**
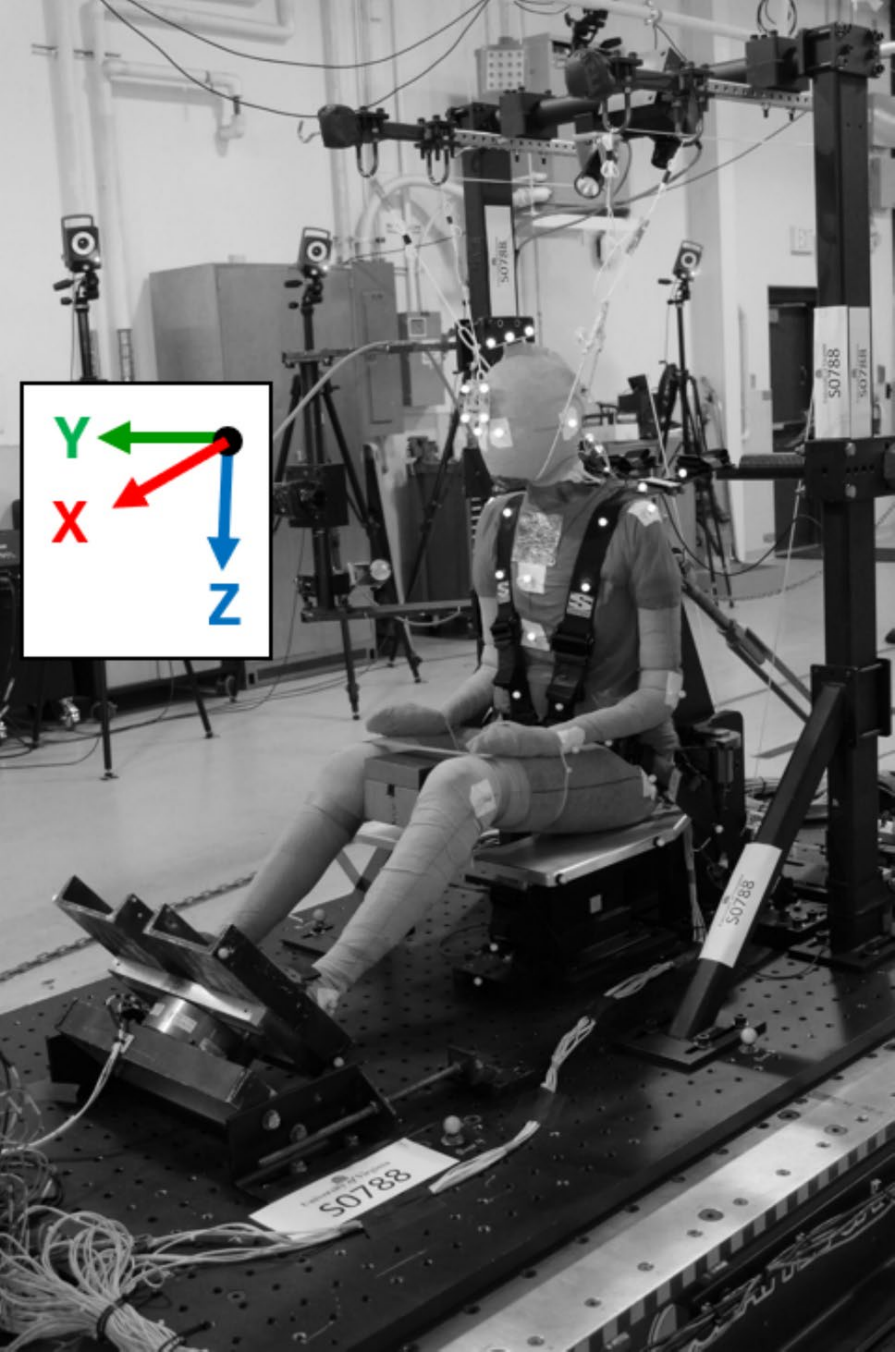
PMHS in fully upright seated posture with 5-point harness restraint on buck mounted to reverse acceleration sled.

**Fig. 2:**
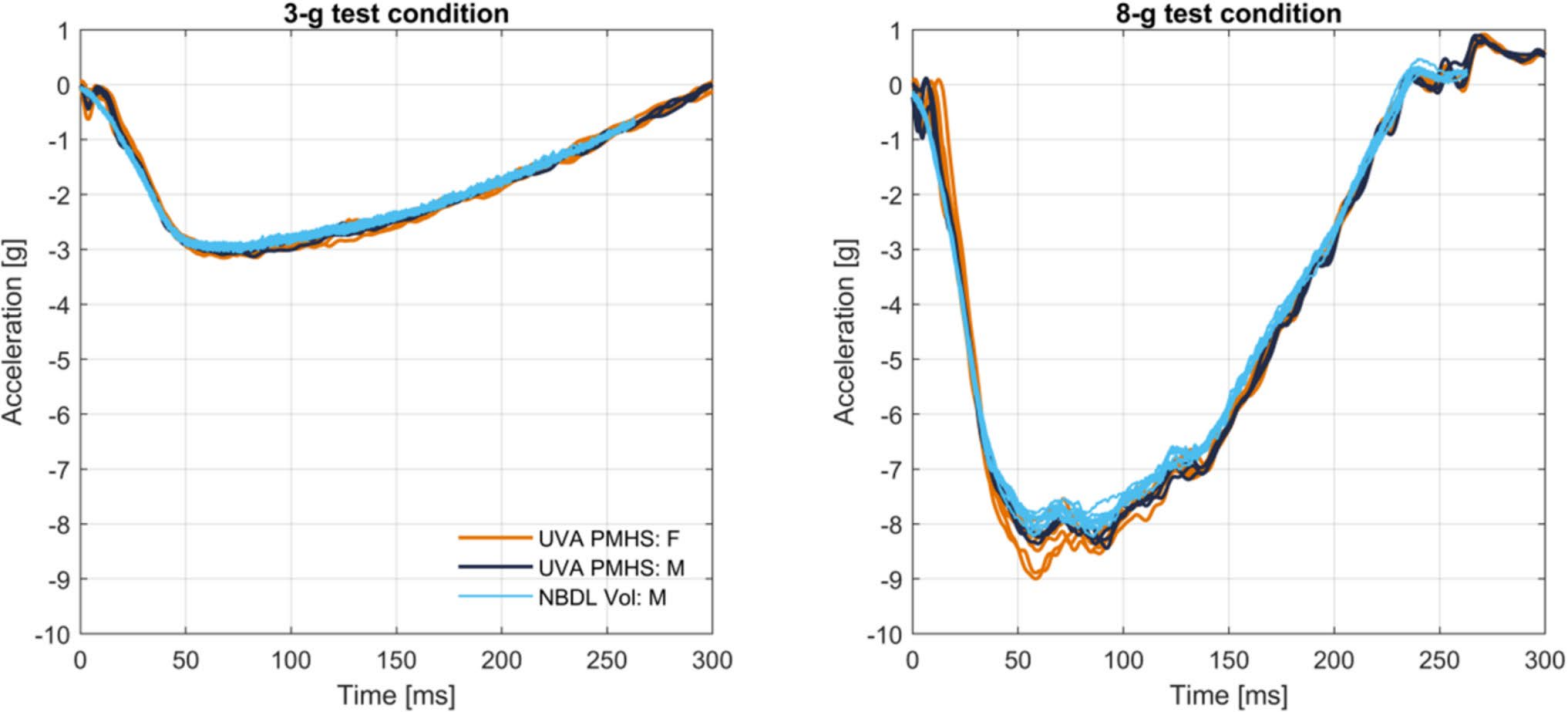
Sled pulses for the 3-g (top) and 8-g (bottom) tests, with acquired sled acceleration time histories for the female tests in orange, the male tests in dark blue, and targets from NBDL male tests in light blue.

Tests were performed on a reverse acceleration sled (Seattle Safety 1.4 MN ServoSled®, Kent, WA, USA) with a test platform or “buck” attached (Figure 1). The buck coordinate system polarities followed Society of Automotive Engineers (SAE) recommendations [40]. The two acceleration pulses (3-g, 20 km/h and 8-g, 43 km/h) were derived from selected NBDL tests (Figure 2; Appendix A). The restraint environment on the buck reproduced the NBDL test setup, with a flat rigid seat bottom, a vertical rigid seatback, and a five-point harness with rigid anchor points (P/N IMR-53119222, Summit Racing Equipment, Tallmadge, OH, USA). Harness anchors were attached to the buck and not to the seat or seatback. Anchor positions were adjusted for each PMHS to provide consistent webbing take-off angles (Appendix B).

PMHS were thawed at 70°F for five days before preparation for the test. Preparation included anthropometric measurements, including neck circumference (Table 3) and surgical installation of instrumentation mounting hardware. After preparation, a CT scan was performed to document the locations of instrumentation mounts. PMHS were stored at 40°F for 36 hours between preparation and testing. Testing was completed within 36 hours in a 70°F laboratory space. At the start of testing, the neck of the PMHS was gently exercised in flexion, extension, torsion, and lateral bending. PMHS were clothed in a cotton shirt and shorts and extremities were wrapped with Coban^TM^ (3 M, St. Paul, MN, USA) self-adhering wrap to contain fluids and support instrumentation cables.

**Table 3:** PMHS head and neck anthropometry.

	UVA subject ID	Neck circumference [mm]	Head to T1 distance [mm]	Head mass [kg]
Small female	1046F	365	140	3.2
	1042F	305	142	3.0
	1048F	300	156	2.7
	1051F	295	161	2.9
	1054F	290	147	2.6
	1049F	355	143	3.1^a^
	Mean	318	148	2.9
	St. dev.	33	8	0.2
Midsize male	1032M	385	166	3.8
	1038M	370	173	4.2
	1031M	405	179	4.2
	Mean	387	173	4.0
	St. dev.	18	6	0.2

^a^Head mass excluded C1.

The PMHS was positioned so that the torso angle (hip center to shoulder center) matched the vertical posture of the NBDL condition [25, 41, 42] rather than the typical upright motor vehicle occupant posture of 25° reclined from vertical [43]. The harness was tightened to maintain PMHS posture and match the NBDL condition’s harness [42]. Volunteer tolerance for pretensioners of 160 N to 290 N [44, 45] was used as guidance and led to tensions of 200 N ± 30 N in the lap belts, 130 N ± 30 N in the shoulder belts, and removal of slack with no quantitative target in the center lap belt. The upper extremities were constrained to the lower back and thighs to prevent them from swinging up to strike the head.

The head was supported by overhead tethers released at the start of the acceleration pulse. Head (Frankfort plane) angle of 0.9° ± 8.9° and neck angle of − 20.8° ± 6.2° and were targeted to match the “Neck Up, Chin Up” posture in the NBDL tests [41, 42] (Figure S3 Appendix C). Head (Frankfort plane) angle was defined as the angle from horizontal of the plane containing the external auditory meatus (EAM) and lower margin of the eye socket. Following SAE recommendations, rotating the chin up yields a positive head angle. Neck angle was defined as the angle from vertical of the line connecting the EAM and the anterosuperior margin of T1. The position of the anterosuperior margin of T1 was calculated during positioning using the location of the T1 instrumentation and measurements taken during the pre-test, post-preparation CT scan. Following SAE recommendations, moving the head forward yields a negative neck angle. The pre-test, in-position distance between the head reference point and the T1 reference point was calculated post-test from motion-capture measurements (Table 3).

**Fig. 3:**
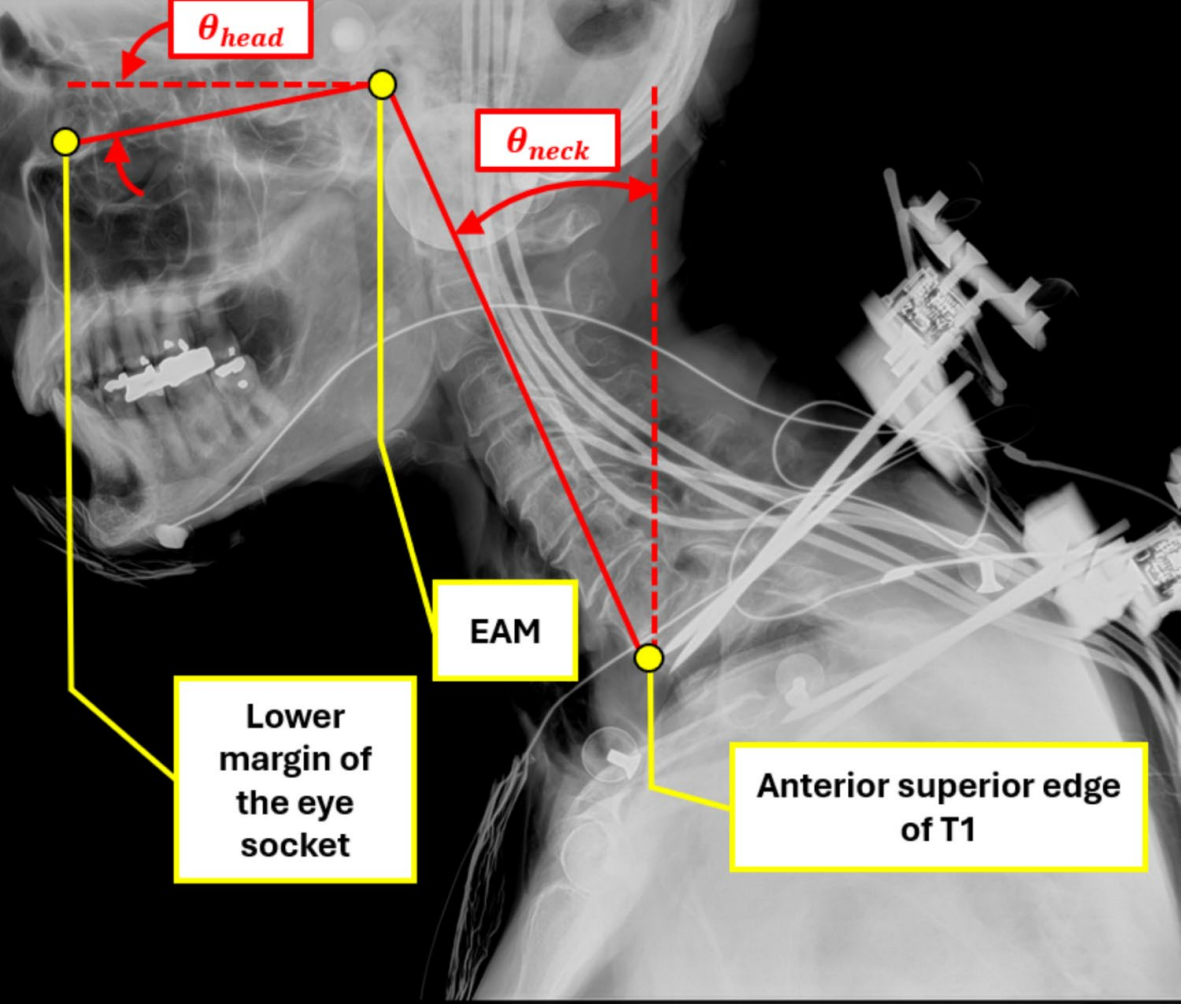
PMHS head and neck angle positioning targets (red, with dashed line representing global horizontal or vertical) defined by anatomical landmarks (yellow) illustrated on an in-position X-ray.

Between the first and second tests for each PMHS, the head and neck were manipulated and X-ray images were captured to check for injury to the neck. After the second test, a post-test CT scan and dissection were performed to identify injuries. A board-certified radiologist analyzed the post-test CT scans and a board-certified surgical technologist (trained in autopsy procedures and injury identification by the Virginia Office of the Chief Medical Examiner) performed the dissection. After dissection and injury documentation, the head mass was measured by separating the head from the body along the plane containing the nuchal and gonion points [46] and transecting the C1–C2 joint space (Table 3). Head mass included C1 (unless otherwise noted) due to difficulty in disarticulating the C1–OC joint. Head mass excluded the tongue (removed along with organ block during autopsy).

An optoelectronic motion-capture system including 20 Vicon TX^TM^ cameras (Oxford, UK) measured at 1000 Hz the motions of retro-reflective spherical markers attached to the buck, the surface of the PMHS, and (in multi-marker arrays) instrumentation hardware affixed to the head and T1 [47]. The motions of the head and T1 were calculated from the motions of the multi-marker arrays using the pre-test CT scan geometry and the assumption of rigid-body motion [48]. The head reference point was defined as the midpoint of the bilateral zygomatic processes and the T1 reference point was defined as the center of the T1 vertebral body [47]. Local coordinate systems consistent with SAE recommendations (SAE J211; [40]) were also defined on the head and T1 (Appendix D). Additional data sources (not included in this paper but available in the NHTSA Biomechanics Test Database) included offboard and onboard high-speed (1000 Hz) videos; seat, seatback, and footpan boundary forces and moments; seatbelt loads; calculated head and T1 accelerations and angular rates; and motions, accelerations, and angular rates of T3.

Corridors representing variation by one standard deviation ellipses and assuming uncorrelated Gaussian variable distribution for sagittal kinematics data were generated using the ARCGen package [49]. For the presented corridors, the optional inputs for the ARCGen package were used: nResamplePoints of 500; nCorrPts of 500; nWarpCtrlPts of 3; warpingPenalty of 0.01; and UseParallel “on.” Corridors were generated from the start of the test (time 0 ms) through the time of maximum Z excursion. By only including data up to the point of maximum Z excursion, the head rebound motion (e.g., return from flexion toward extension) was excluded from the corridors. The corridors were generated based on the cross-plots of the data, not time-history based corridors (Appendix E). The time histories (non-truncated) of relevant kinematics are provided in Appendix F.

## Results

### Subject Positioning

The test-day measures of head and neck angle for the in-position PMHS are provided in Table 4. Additionally, the initial pitch (i.e., the average from − 50 ms to 0 ms) of the T1-based coordinate system in the global coordinate system from post-processed Vicon is in Table 4. On average, the PMHS had a slightly negative head angle (nose tilted slightly down). In contrast, the original NBDL positioning targets were slightly nose up. However, it should be noted that the NBDL reported head angles ranged from − 10.7° to 20.4° [41, 50]. The head angle in the current study was well within this range of variability and was highly consistent within the PMHS tests (st. dev. 2.3° across all tests). For the neck angle, the UVA PMHS mean angle was a few degrees more forward than the NBDL target (− 20.8°), but again, within the range of − 5.5° to − 32.6° reported by NBDL and consistent across this study (st. dev. 3.5°)[50]. The T1 angle for the PMHS was pitched forward by an average of − 41.7°, which can be qualitatively observed in Figure 3. There was no published documentation of T1 pitch from the NBDL studies to the authors’ knowledge.

**Table 4:** PMHS head and neck initial posture determined from post-processed, transformed motion capture data (measures in degrees).

	UVA test number	Head angle	Neck angle	T1 angle
		(θ_head_)	(θ_neck_)	(θ_T1_)
Small female	S0785	− 2.0	− 13.9	− 40.8
	S0786	− 7.3	− 18.8	− 41.0
	S0787	− 5.2	− 28.9	− 49.1
	S0788	− 0.1	− 27.1	− 46.0
	S0789	− 7.7	− 22.6	− 37.9
	S0790	− 4.2	− 21.3	− 37.8
Midsize male	S0791	− 1.6	− 21.1	− 44.1
	S0792	− 0.8	− 23.3	− 47.6
	S0793	− 4.3	− 20.2	− 60.2
	S0794	− 1.2	− 19.1	− 61.2
	S0795	− 1.1	− 26.1	− 36.3
	S0796	− 3.4	− 23.9	− 38.2
Small female	S0797	− 3.2	− 24.7	− 29.8
	S0798	− 0.9	− 25.4	− 23.9
	S0799	− 0.6	− 20.5	− 35.4
	S0800	− 0.8	− 21.3	− 37.7
	S0801	− 2.1	− 23.7	− 41.7
	S0802	− 0.8	− 23.1	− 41.4
Average		− 2.6	− 22.5	− 41.7
St. dev		2.3	3.5	9.2
Max		− 0.1	− 13.9	− 23.9
Min		− 7.7	− 28.9	− 61.2

## Qualitative Description of Resulting Kinematics

**Fig. 4:**
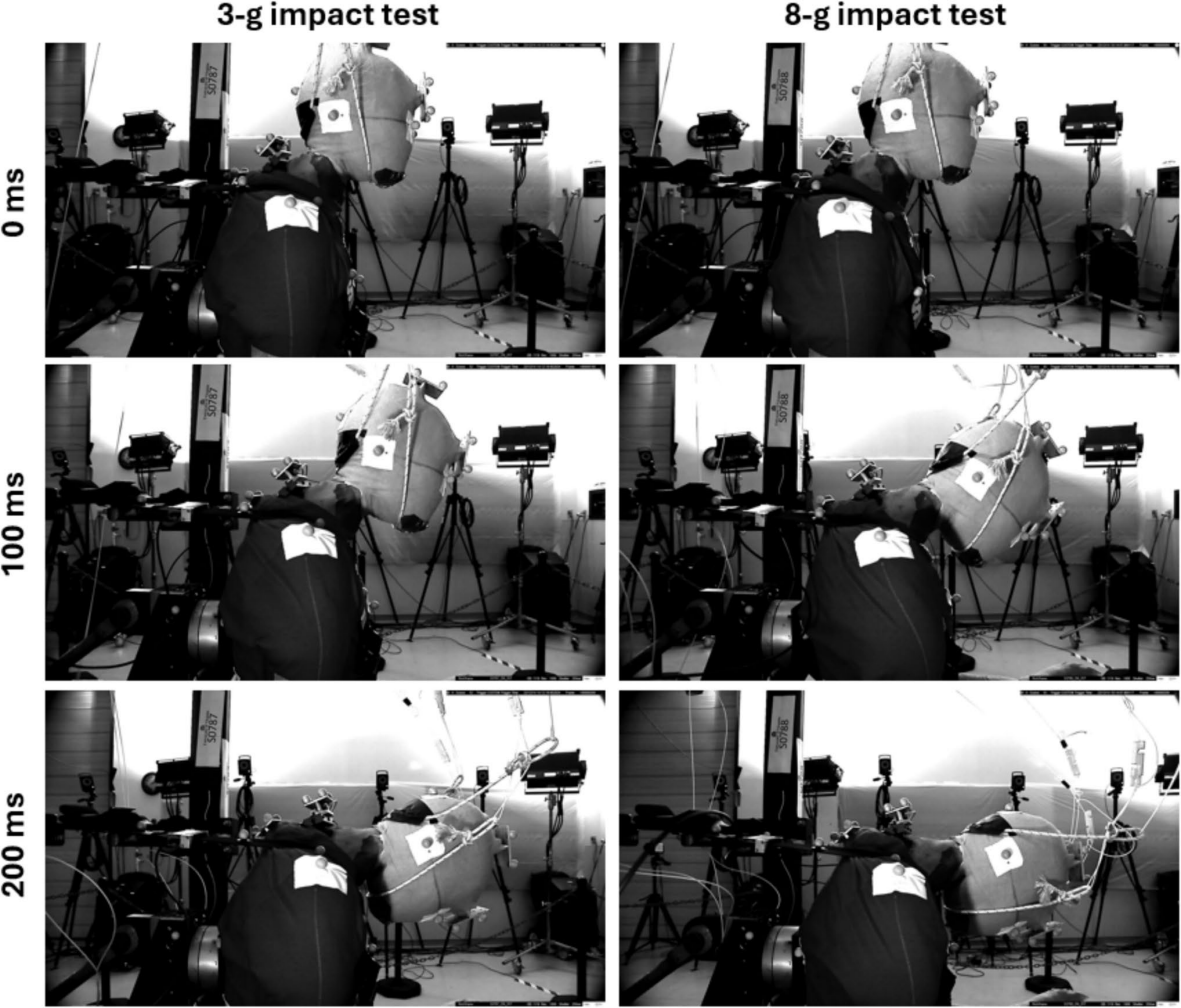
Example PMHS motion at 0 ms (top), 100 ms (center), and 200 ms (bottom) from a high-speed video camera mounted on the sled to the right of the same PMHS for the 3-g impact test (left) and 8-g impact test (right).

An example of the overall motion of the head and neck is illustrated in Figure 4 for a 3-g and an 8-g impact test with a representative PMHS. At 0 ms, the similarity of head and neck postures for two different tests with the same PMHS shows the consistency of initial measurements as noted previously in Table 4. In both 3-g and 8-g impact conditions, the first part of the motion sequence (up to around 100 ms) is primarily forward translation of the head with minimal movement of T1. In the 8-g condition, this first part of the motion sequence also includes forward rotation (negative pitch) of the head. Up to about 100 ms, the PMHS torso has moved relative to the shoulder harness straps by sliding forward. After about 100 ms, the PMHS is fully engaged with the harness restraint. In both 3-g and 8-g impact conditions, the next part of the motion sequence (up to around 200 ms) is forward rotation and downward translation of both the head and T1. At 200 ms, there is more downward translation of the head and T1 for the 8-g impact condition compared to the 3-g impact test.

## T1 Acceleration Compared to NBDL Data

**Fig. 5:**
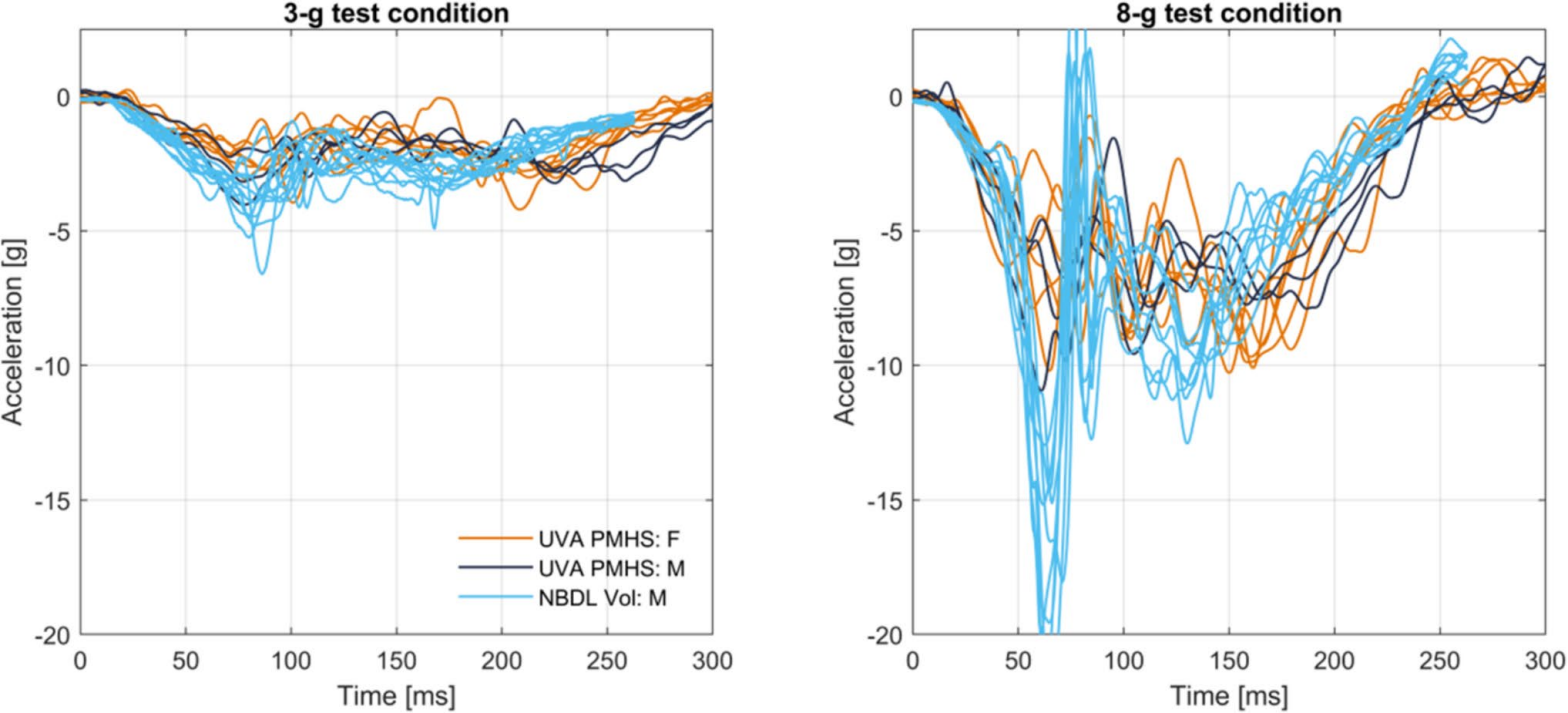
T1 acceleration in X for the UVA PMHS and original NBDL male volunteers for the 3-g (left) and 8-g (right) test conditions.The small female PMHS are in orange, the midsize male PMHS are in dark blue, and the midsize male NBDL volunteers are in light blue.

The forward (X) T1 accelerations for the PMHS and original NBDL volunteer data are in Figure 5. There is no clear separation of small female and midsize male PMHS accelerations. The PMHS T1 accelerations are similar to the peak sled accelerations, indicating minimal relative motion between the PMHS torsos and the buck, thus isolating neck flexion from variations in motion below the neck. The accelerations of the NBDL volunteers and PMHS were similar at the lower severity 3-g test condition. The NBDL volunteer peak T1 accelerations exceeded the PMHS peak accelerations in the 8-g testing condition. Given the observed and documented motion of the T1 surface instrumentation for the NBDL volunteers [25], there may be more artifactual motion of the NBDL T1 traces compared to the bone-mounted T1 instrumentation used with the PMHS.

## Kinematics

**Fig. 6.**
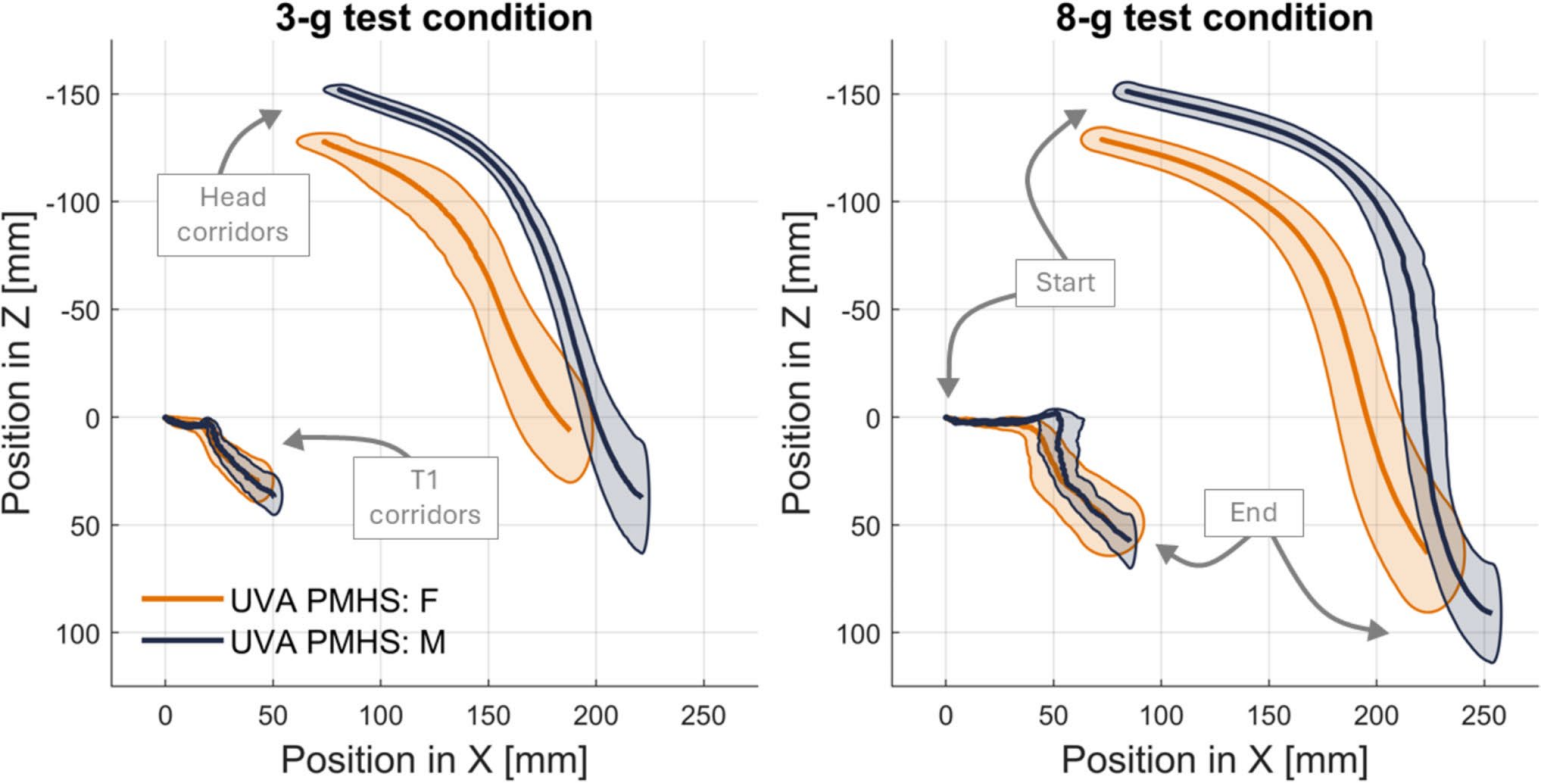
With the origin at the initial position of T1, the sagittal plane (X–Z) head position corridors (characteristic average ± 1 standard deviation) for the 3-g (left) and 8-g (right) test conditions. The small female is in orange and the midsize male is in dark blue.

**Fig. 7:**
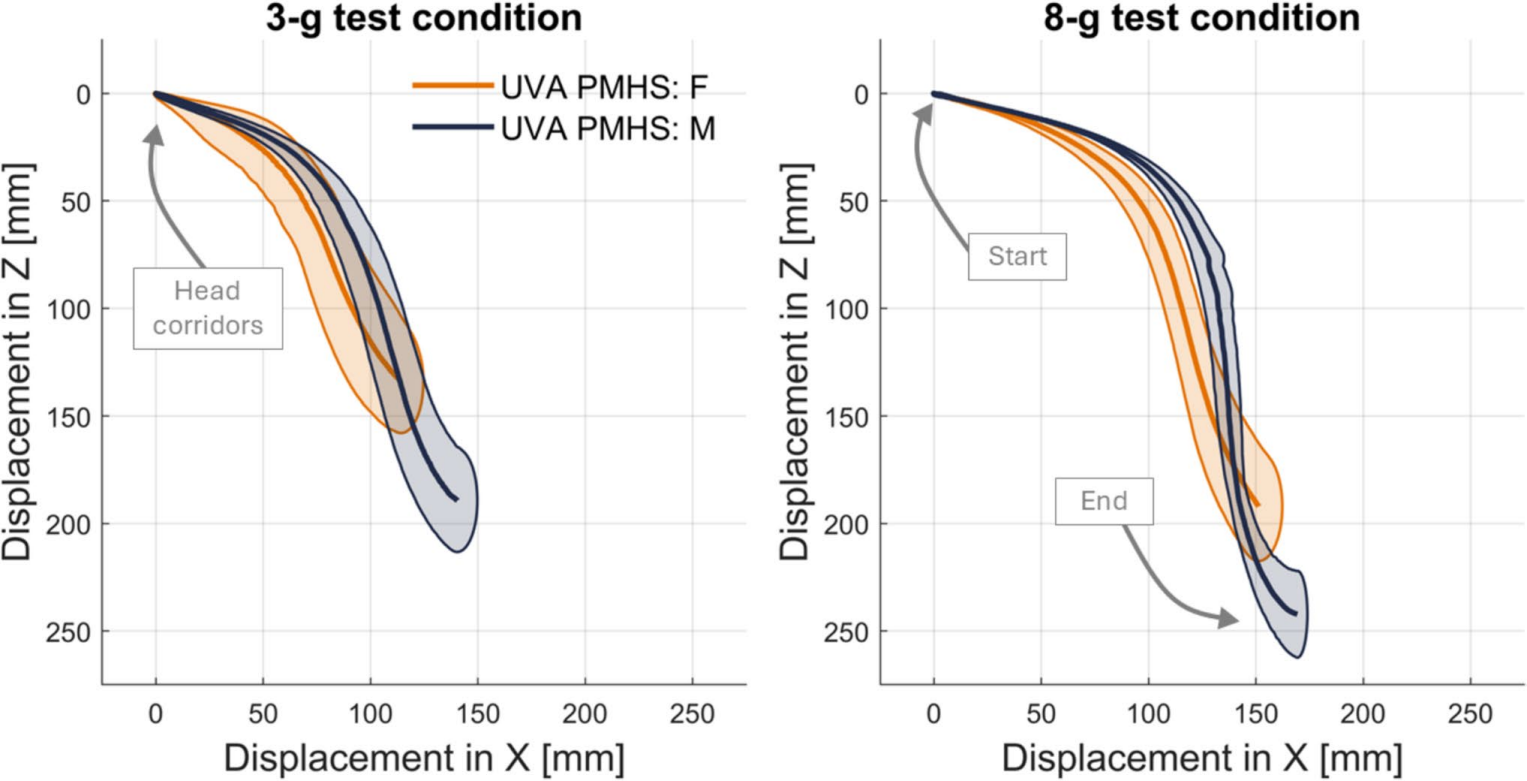
With the origin at the initial position of the head, the sagittal plane (X–Z) head displacement corridors (characteristic average ± 1 standard deviation) for the 3-g (left) and 8-g (right) test conditions. The small female is in orange and the midsize male is in dark blue.

Two kinematic measures were examined to assess sagittal (X–Z) plane motion at both impact severities (3-g and 8-g) for both PMHS groups (small female and midsize males). First, the linear displacements of the head and T1 with respect to the buck were assessed based on motion-tracking data that were transformed to the skull and vertebra (Figure S4 Appendix D; Figure 6 and Figure 7). In these figures and in accordance with the SAE coordinate system, X translation represents horizontal motion (positive is forward) and Z translation represents vertical motion (positive is downward). The sagittal displacements in Fig. 6 show increasing magnitude of excursions when increasing impact severity from 3-g to 8-g. Starting with the T1 kinematics, both females and males exhibit a bilinear response, starting with nearly completely horizontal forward translation, followed by combined forward horizontal and downward translation. The total horizontal motion of T1 was an average of 44 mm and 52 mm for females and males, respectively, for the 3-g impact tests, and an average of 77 mm and 87 mm for females and males, respectively, for the 8-g impact tests. The magnitude of T1 vertical motion was smaller than the magnitude of T1 horizontal motion for all tests. The corridors for T1 excursion are similar between small females and midsize males for both impact severities, although there was slightly more variation in the 8-g impact than the 3-g impact.

In Fig. 6, the head of the males starts higher (more negative in Z, by an average of 23 mm) and more forward (more positive in X, by an average of 9 mm) relative to T1 compared to the females, which naturally follows for the midsize male (taller) compared to small female (shorter) anthropometry. Figure 7 debiases the head motion (e.g., move the origin to the head, rather than the T1 used in Fig. 6), which allows for a more direct comparison for head motion between small females and midsize males. Like T1, the head motion shows a bilinear response that starts with predominately forward horizontal excursion and is followed by predominately vertical downward excursion. This bilinear head motion response is more pronounced in the 8-g impact test compared to the 3-g impact tests. In Fig. 7, the small female and midsize male head motions, illustrated as starting from the same location, are similar in trajectory, with the males exhibiting a larger magnitude excursion, especially in the 3-g condition.

The mean (and standard deviation) peak measures for head motion are provided in Table 5. These average peak values were compared across small females and midsize males, as well as across the 3-g and 8-g impact severities in Table 6. The decreases in peak motion from 8-g to 3-g were greater for small females than midsize males. The largest change from 8-g to 3-g was for the Z linear displacement of the head, where small females and midsize males showed a 30% and 22% decrease, respectively, in peak excursion. When comparing the decrease in peak motion from midsize males to small females, the largest differences were seen in the 3-g tests.

**Table 5:** Peak kinematic measures for head motions for each anthropometry group (small females and midsize males) and each test condition (3-g and 8-g impact). Provided as an mean (st. dev.).

	Small female		Midsize male	
Measurement	3-g	8-g	3-g	8-g
X linear displacement [mm]	114.2 (11.5)	151.3 (12.0)	140.4 (11.5)	169.3 (5.7)
Z linear displacement [mm]	134.2 (26.0)	192.1 (27.8)	189.4 (29.3)	242.3 (24.8)
Pitch [deg]	− 68.5 (11.9)	− 83.0 (10.4)	− 78.2 (8.8)	− 90.2 (7.3)

**Table 6:** Decreases in mean peak head motion measures for different anthropometry groups and test conditions.

	Decrease from 8-g to 3-g		Decrease from midsize male to small female	
Measurement	For females	For males	For 3-g	For 8-g
X linear displacement [mm]	− 25%	− 17%	− 19%	− 11%
Z linear displacement [mm]	− 30%	− 22%	− 29%	− 21%
Pitch [deg]	− 17%	− 13%	− 12%	− 8%

**Fig. 8:**
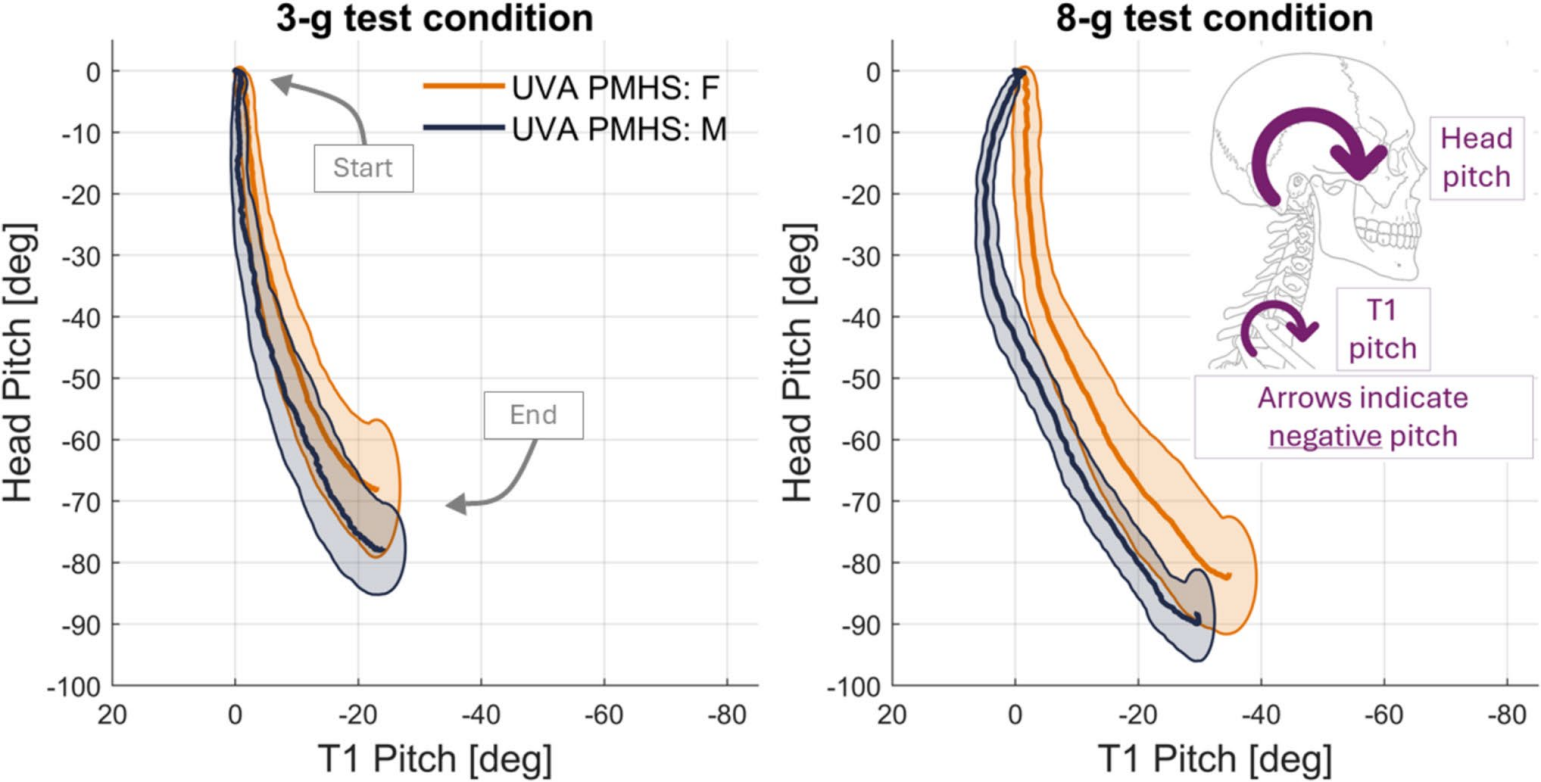
The debiased pitch corridors (characteristic average ± 1 standard deviation) for the head and T1 for the 3-g (left) and 8-g (right) test conditions. The small female is in orange and the midsize male is in dark blue. Negative pitch is forward rotation, and positive pitch is rearward rotation (diagram on right).

The second kinematic measure assessed in the sagittal plane was the rotation of the head and T1. Specifically, the pitch (rotation about the Y-axis in the X–Z plane) of the transformed motion-tracking data for the head and T1 was examined in Fig. 8. In this figure and in accordance with the SAE coordinate system, negative pitch represents forward rotation (chin-to-chest motion). Here, both the rotation of the head and T1 are with respect to the buck. Figure 8 illustrates how in (or out) of sync the rotation of the head and top of the torso (represented by T1) are. For the PMHS tested, the head rotated with almost no change in T1 pitch at the start of the motion. After about 40° of head forward rotation, there is combined head and T1 rotation, as captured with on-board video in Fig. 4. The pitches in Fig. 8 are similar for small females and midsize males at 3-g in terms of overall trajectory, magnitude, and variability between subjects. The difference between female and males for T1 pitch was only an average of 1.6°. Despite the similarity of pitch at T1 at 3-g, midsize males exhibited 9.5° (12%) more head pitch compared to small females (Table 6). Compared to 3-g, at 8-g, there was more separation of small female and midsize male pitch responses, especially at the initiation of motion. At T1, pitch for males started with slight rearward rotation of up to 6.7°, followed by forward rotation (Fig. 8). In contrast, T1 motion for small females started and continued with forward rotation until rebound. For peak T1 pitch at 8-g, males exhibited lower absolute peak pitch compared to small females (by 5°), although the range (difference between minimum and maximum) of T1 pitches for females and males were similar (on average, 36° for females and 35° for males). For both females and males at 8-g, the head exhibited forward rotation until rebound, with average peak head pitch of 83° and 90° for small females and midsize males, respectively.

## Injuries of PMHS

Autopsies were performed at the conclusion of all testing for each PMHS to identify injuries, with a focus on musculoskeletal injuries to the cervical spine (Table 7). No injuries were noted for the midsize male PMHS. For small females, two of the six PMHS had neck injuries and three of the six PMHS had rib fractures. There was a lower extremity fracture found for one PMHS (1051F).

**Table 7:** Injuries corresponding to each subject and test.

UVA subject ID	UVA test number	Diagnosed injuries
1046F	S0785, S0786	Right C2–C3 partial facet joint disruption
		C6–C7 partial (nearly complete) interspinous ligament tear
		C7 superior–posterior endplate fracture
1042F	S0787, S0788	Right 2^nd^ rib anterior bi-cortical non-displaced fracture (105 mm from sternum along rib)
1048F	S0789, S0790	Right 2^nd^ rib anterior bi-cortical non-displaced fracture (90 mm from sternum along rib)
		Left 2^nd^ rib anterior bi-cortical non-displaced fracture (145 mm from sternum along rib)
		Left C4–C5 partial facet joint disruption
		Right C7–T1 partial facet joint disruption
1032M	S0791, S0792	None
1038M	S0793, S0794	None
1031M	S0795, S0796	None
1051F	S0797, S0798	Right 1^st^ rib anterior bi-cortical non-displaced fracture (50 mm from sternum along rib)
		Left femur greater trochanter fracture
1054F	S0799, S0800	None
1049F	S0801, S0802	None

## Discussion

### Comparison to NBDL Original Testing

The tests presented in this study are the first to imitate NBDL conditions with small female PMHS. The reverse acceleration sled system used was able to match both the 3-g and 8-g input accelerations from the original testing (Fig. 2). Based on qualitative descriptions in text and photos of the NBDL testing set-up, the seat and restraint used for PMHS testing are similar [25, 41, 42]. The posture of the PMHS matched the NBDL volunteers, specifically the “neck up chin up” positioning quantified by head and neck angles (Figure S3; Appendix C) [41, 42].

In the current study, the T1 acceleration magnitude of the midsize male and small female PMHS in the 8-g tests was less than that observed in the NBDL volunteers (Fig. 5). Contemporaneous testing of midsize male PMHS observed the same difference at a higher severity (15-g) impact compared to volunteers [31]. In both cases of PMHS testing, the difference in T1 acceleration from volunteers may be due to actual differences in torso motion or artifactual peak in the volunteer data from instrumentation package slippage relative to the subject.

Differences in torso motion could be the result of differences in harness tightening, subject positioning, or the presence/absence of bracing. To the authors’ knowledge, there was no methodological quantification on the harness tightening procedure for the original NBDL testing. In the PMHS tests, the shoulder belt segments were pretensioned to 125 N ± 15 N and the lap belt segments were pretensioned to 204 N ± 23 N. Overall, the positioning and pre-tensioning were symmetric between the right and left sides of the PMHS and consistent across the test series. An inclusive list of pre-test measurements is in the NHTSA report [50]. These data can be used as target boundary conditions for replications or simulations of the PMHS tests.

The PMHS tests used 3-g and 8-g input accelerations, which are a sample of the available frontal testing conditions captured by NBDL [42]. The 15-g (60 km/h) test condition has been used previously in biomechanics literature [1–3, 5, 25, 26, 31], as it presents the most severe condition available from NBDL. The 15-g was not selected for this test series with small females, where biofidelity target kinematics unaltered by catastrophic injuries were desired. There was evidence from previous testing in an NBDL-like condition with PMHS [31] that neck injuries could occur in males and females at 15-g. Additionally, previous testing with small females and midsize males (NHTSA Biomechanics Database, Test Numbers 11491–11494; 11509–11511; 12803–12807) showed that neck injuries could occur starting around 9-g (30 km/h) with a 3-point belt with a load limiter. Given the injuries observed in Table 7 from post-test dissection, it is likely that, if the small female PMHS had been tested up to 15-g, neck injuries would have occurred with greater frequency and severity than what was observed at 8-g.

Past studies in NBDL conditions have observed that maximum head rotation and maximum head displacement of PMHS are greater than those of volunteers [28, 51–54]. Wismans et al. reported an average maximum head rotation for the 8-g frontal NBDL condition a little over 60° [52]; Correria et al. reported at peak head rotation around 70° for NBDL volunteers in the 8-g condition [53]. Both indicate that the midsize male PMHS peak value of 90° (Table 5) exceed the volunteer head rotation. Studies on the GHBMC also assessed forward head displacement for the NBDL 8-g testing condition, showing an average peak displacement around 140 mm [28, 53]. Similar to head rotation, the PMHS maximum values for midsize males at 8-g exceed the volunteer magnitude. The midsize male PMHS tested here and the original NBDL midsize male volunteers will be directly compared in future analyses. Overall, comparisons with published NBDL results indicate that the excursion and head rotation of the presented PMHS corridors (for the midsize males) are an overestimate of the living response. These variations may be due to procedure, subject variability, muscle tone, or a combination. Regardless of cause, PMHS response may be used as an upper bound for biofidelity assessment of physical and computational surrogates.

The Wismans et al. data [31] showed that head excursion and rotation of PMHS exceed volunteer envelopes; it is reasonable to expect that the corridors presented here of PMHS data may be an overestimation of the magnitude of the volunteer response. Wismans et al. successfully collected data for two female PMHS, whose anthropometry was smaller in weight and smaller or similar in height compared to the five male PMHS tested. The resulting female sagittal excursion was smaller than male excursion for both the 15-g and 23-g tests, with similar head rotation. The results presented in this paper show the same difference, though the PMHS tested differed in both sex and target anthropometry (e.g., analysis was between midsize male and small female).

## Analysis of Kinematics

The T1 kinematics in Fig. 6 highlight how well the PMHS were coupled to the sled. This was a goal in this study for replicating the NBDL methodology to isolate neck flexion from confounding motions of the rest of the body in a sled impact. The initial horizontal motion of T1 corresponds to the top of the PMHS torso moving forward at the beginning of the sled pulse, before the harness fully engages with the subjects. This motion reflects the physical boundary conditions of this test series, given the fully upright posture of the PMHS and horizontal aluminum seat pan. The initial horizontal motion of T1 was most pronounced for the midsize male corridors for the 8-g impact, which is expected for the higher acceleration test with the higher mass PMHS group. After harness engagement, there was a small amount of downward and forward translation of T1, as the PMHS pitched slightly forward, moving slightly with respect to the shoulder portions of the harness.

Figure 6, with the origin at the initial position of T1, appears to illustrate the small female head motion corridors separated from the midsize male corridors; this is because small females are smaller in stature than midsize males. It is useful in illustrating that for the same environment and target seated posture examined in this study, small females will exhibit a smaller envelope of space with resulting head excursions. Figure 7 assesses the differences in displacement of the head, rather than the differences in position as shown in Fig. 6. In Fig. 7, small females still have smaller magnitude excursions forward and downward, but there is overlap in small female and midsize male corridors. This smaller arc from small females still relates to anthropometry; small females have a shorter head-to-T1 distance and therefore a smaller radius of rotation about T1. The anthropometry-driven differences between small female and midsize male excursions has also been observed in volunteers [55]. As noted in Table 6, there were smaller differences between small females and midsize males at the 8-g test compared to the 3-g test. This suggests that even in the passive response captured by PMHS (i.e., with no tensing musculature), there may be sensitivity to lower impact severities that varies by occupant size.

The rotation of the head was fairly similar between small females and midsize males for both 3-g and 8-g tests, but the corridors diverge for the two anthropometries at 8-g due to the differences in T1 pitch (Fig. 8). Midsize males exhibited initial rearward rotation of T1, which was not observed in the female data. There could be several factors contributing to this difference, including variations between the sexes in boundary conditions or mass distributions. Despite the initial rearward rotation at T1, midsize males still exhibited larger maximum forward head pitch than small females (as well as linear excursions). This indicates that for impact-related range of motion, larger anthropometries still exhibit larger magnitudes of head motion. The difference in head mass between the midsize male and small female (4.0 kg ± 0.2 kg and 2.9 kg ± 0.2 kg, respectively) was a contributing factor in the inertial loading observed in this test series.

The analyses described above have used summary measures, like peak excursion, to generally compare small female and midsize male responses, as well as variations based on impact severity (e.g., 3-g versus 8-g). Along with the corridors in Figs. 6 and 7 and high-speed video available, descriptions and contrasts of the motion are possible. However, additional methods to investigate the differences of curve shape may be insightful, especially where qualitative variation may be observed (e.g., female versus male T1 pitch at 8-g). Such methods include curve-based analyses such as statistical parametric mapping [56] or functional data analysis based regression [57].‬

Figure 8 shows the sagittal pitch of the head and T1 each starting at 0° at t = 0, which facilitates comparisons and corridors of the curves for time after zero. The initial position of the head and T1 were not 0°: female head and T1 pitch started at an average of − 4.6° and − 38.5°, respectively, while male head and T1 pitch started at an average of − 7.0° and − 48.0°, respectively. The initial neck angle (from the T1 to the head), was, on average, − 29.6° for females and − 28.6° for males. Note that the head, T1, and neck angles derived from calculated internal skeletal motions differ from the values listed in Table 4 for head (e.g., Frankfort) and neck angles, which were based on external landmarks. In the use of the corridors presented here, the initial posture of the head and neck may be important factors to replicate and consider in model or surrogate implementation.

The initial phase of motion (before saturated harness engagement) for both small female and midsize male PMHS showed greater magnitude head pitch than T1 pitch; T1 motion lagged behind head motion for pitch. In contrast, the original NBDL experiments have typically observed the opposite phenomenon, illustrated by “head lag” plots [1, 25]. Head lag plots show the neck link (rigid link connecting T1 to the OC) rotation and head rotation for midsize male volunteers, where there is about 30° of neck link rotation prior to any substantial head rotation in a 15-g impact pulse [25]. In a previous analysis [58], the pitch of the head and T1 at 3-g and 8-g for NBDL data was examined and compared to the UVA PMHS testing, finding a slight head lag in head-T1 pitch plots for NBDL data, though to a lesser extent than the head link lag presented in Thunnissen 15-g impact pulse. The differences between UVA PMHS and NBDL volunteer data may be due to differences in test set-ups and boundary conditions (e.g., restraint tension, restraint material, seat material/friction, detailed torso positioning), subject type (e.g., volunteer versus PMHS), and the quality of T1 instrumentation data (e.g., surface versus bone mounted, sampling rate).

While the original NBDL dataset is irreplaceable and unrepeatable, some of the original data, namely the T1 instrumentation, is flawed because the attachments to the volunteers were questionable. This has led to retrospective data corrections [25]. Here, because we were testing with PMHS, we were able to mount instrumentation and Vicon marker arrays rigidly to the bones. This avoided shifting of the data tracking packages relative to the subject, which was the concern with the original NBDL T1 data. This provides clean boundary condition data at T1 for an NBDL-like testing setup using PMHS, which can be used as inputs for validation efforts. Specifically, T1 kinematics can be used for component-level assessments of head and neck models (e.g., a sectioned computational model or dummy). Component-level testing of head and neck models have used horizontal (X-direction) acceleration of the base of the neck as the input and the resulting motions of the head and neck as the output [1]. The X-acceleration of T1 from the NBDL testing is the standard input for such component-level testing. This simplification, however, may not suffice, since it excludes any vertical motion of T1 and (perhaps more importantly) it excludes any rotational motion of T1. The Z excursion of T1 was, on average, 69% the magnitude of X excursion and the pitch was an average (across females and males) of − 24° for the 3-g impact tests and − 35° for the 8-g impact tests. These motions should be addressed, in addition to the X-direction loading, when modeling inputs for T1. In a HBM, this can be implemented by prescribing multiple boundary condition kinematics. In physical testing, dynamic, multi-axial testing rigs would be required to fully replicate the PMHS-derived T1 kinematics.

## Analysis of Injuries and Other Post-test Measures

Injuries were found for some of the small female PMHS following the completion of the 3-g and 8-g impact tests; these injuries were mainly to the cervical spine and ribs. Based on the lack of injuries observed in this inter-test (between the 3-g impact and 8-g impact) examination, it is reasonable to assume that the injuries found during post-test PMHS dissection (Table 7) occurred during the 8-g impact tests, and not the 3-g impact tests.

Two of the small female PMHS had injuries spanning multiple levels of the cervical spine and conceptually consistent with neck flexion. The most common type of neck injury was partial facet joint disruption (at C2–C3 for 1046F and at C4–C5 and C7–T1 for 1048F). Additionally, 1046F had a nearly complete interspinous ligament tear between C6 and C7. Neck flexion could put the facet joint capsules and posterior ligaments into tension, resulting in these injuries. Looking into potentially related factors, including those reported in the posted report with the tests on the NHTSA Biomechanics database [50], PMHS 1046F did have the largest body mass, head circumference, and neck circumference for the small females tested, while PMHS 1048F was below the small female averages for these three measures, though not the smallest reported. 1046F and 1048F (along with 1054F, which did not have any injuries reported) exhibited the highest peak magnitude linear (X) and angular (Y) displacements for the 8-g impact test, as well as calculated moment about the occipital condyles. Overall, the PMHS with facet capsule injuries were in the more extreme half of small female kinematic responses. In addition to the facet and ligament injuries, PMHS 1046F also had a C7 endplate fracture, located on the superior–posterior aspect of the vertebral body. Based on pre- and post-test CT, as well as post-test PMHS dissection, the mount screws placed in the T1 vertebral body for instrumentation for 1046F were centered in the vertebral body; they were unlikely to have caused the superior endplate injury of C7 as they did not bridge the C7–T1 disk nor link the C7 vertebral body to the C7 superior endplate. This endplate fracture may have occurred during the rebound and neck extension phase (400 to 500 ms). Similarly, facet joint capsules and spine ligaments were unlikely to be affected by instrumentation.

The observed cervical spine fractures are likely due to the individual neck kinematics and kinetics (both subjects exhibited large peak head kinematics relative to the cohort) and PMHS factors (e.g., whole body and regional neck anthropometry, tissue material differences); it is unlikely that they are related to the instrumentation placed at T1 or other testing-related handling. The neck injuries that were observed were indicative of initiation of injury (e.g., mostly partial disruptions or tears) and were not catastrophic. The small amounts and minor severities of neck injuries validates the approach of only testing to a maximum sled pulse of 8-g, rather than the maximum sled pulse of 15-g endured by NBDL midsize male volunteers, to avoid severe injuries that could have added more complexity to the resulting kinematics and kinetics. However, the occurrence of even minor or initiating injuries must be considered when interpreting the kinematic data presented here.

This study tested each PMHS twice in whole-body acceleration sled impacts following precedent from past kinematics studies [59–61]. The limitation of repeated testing on PMHS is that injuries may occur which affect PMHS response in subsequent tests. The study design aimed to minimize the risk of severe damage (e.g., vertebral fractures, complete ligament tears) by selecting a low-speed condition as the first test. It is possible that the observed injuries occurred in the low speed test; however, since not all subjects suffered injuries, it is also possible that the observed injuries occurred in the high-speed tests. After the first test with each PMHS, no gross abnormality of head and neck flexibility was observed by palpation and manipulation, and no difference in bony structures were observed by X-ray imaging. Therefore, severe bone and joint failures that would affect head and neck motion can be reasonably excluded.

Observed neck injuries of two of the female PMHS after the 8-g test from autopsy were partial (unilateral) facet disruptions (for both 1046F and 1048F), as well as a nearly complete interspinous ligament tear and superior endplate fracture (1046F). Because these neck injuries were not severe, they would have been difficult to detect with the methods used between the 3-g and 8-g test (e.g., palpation and planar X-ray). If injuries did initiate in the 3-g test, they could influence the kinematics of the 8-g test (discussed in more detail below) and the injury itself would worsen. For instance, the nearly complete interspinous tear observed during autopsy for 1046F may have started as a partial ligament tear at 3-g and progressed to a nearly complete tear at 8-g.

It is possible that the neck injuries suffered by two of the nine PMHS (1046F and 1048F) occurred in the first (low speed) test, which may have affected the kinematics of those two PMHS in the second (high speed) test. The most likely effect would be to increase the compliance of the neck in flexion (and, to a lesser degree, in tension) and therefore to increase the rotation and displacement of the head relative to the neck. Since the two PMHS (1046F and 1048F) who experienced injuries already showed the highest displacement and rotation of the female PMHS in the high-speed (8-g) test, this means that if the injuries affected the kinematics, and then those two PMHS acted as a slightly higher upper bound for small female response. Therefore, the data generated in this study are valid for use as biofidelity reference targets.

In addition to the cervical spine injuries described above, there were a few observed skeletal injuries to other body regions. Three of the small female PMHS had one or two upper, anterior rib fractures after the 8-g (43 km/h) test. The rib fractures were about 10 cm lateral from the sternum, which, based on pre-test measurements of harness positioning, places the fractures near the shoulder portions of the harness. The three small female PMHS with rib fractures, 1042F, 1048F, and 1051F, were the oldest in age of the small females at 75, 71, and 73 years, respectively. 1042F and 1048F had the lowest bone mineral density of the small female PMHS tested (1051F had the best spinal bone mineral density of the cohort, measured L1–L4). 1051F, in addition to a rib fracture, had a fracture on the greater trochanter of the left femur. This subject was the tallest female PMHS (1690 mm) and did have atypical positioning of the torso [50]. Specifically, because of the narrowness of the seatback, the shoulder belt portions of the harness adducted the scapula so that the shoulders were more drawn back than with other subjects. Upon review, the PMHS mass, PMHS age, bone mineral density, leg positioning, and lap belt forces for 1051F and the associated sled runs were similar to the other tests in this series. It is unclear, with the available data, how or when this fracture occurred.

The NBDL testing condition can be used to help understand the head-neck response and assess the kinematics of injury prediction tools (e.g., crash test dummies, computational models), but the loading mode and resulting injuries are not representative of an automotive environment. Here, the PMHS were seated fully upright with the Frankfort plane near zero (eyes forward-facing and level). In current automotive assessments, the seats have some amount of recline (25°, for example), with an eyes-facing forward head position [62], resulting in initial boundary conditions and loading modes of the cervical spine that may differ compared to the NBDL configuration presented in this test series. Despite this, however, the NBDL-like test condition still provides valuable information as the results can be combined with the large-scale volunteer dataset of the NBDL to bridge between PMHS and volunteer responses.

The head mass included in Table 3 provides estimates for both midsize males and small females. These mass measurements do not include the tongue or neck structures continuous with the thoracic organ block, as these were removed earlier during the post-test autopsy. The head mass included C1 (except for 1049F) due to the relative accessibility of C1–C2 for transection compared to the junction of the OC and C1. During test preparation, testing, and post-test autopsy, minimal to no brain matter, blood, nor cerebrospinal fluid (CSF) loss was observed through any cranial orifices nor instrumentation sites.

For the midsize males in this study, the average recorded head mass was 4.0 kg, which is a slight underestimate compared to previous predictions (4.1 kg [63, 64]). The THOR-50 M head is slightly heavier than the Schneider target, coming in at 4.54 kg [65]. The head mass of the midsize male PMHS was comparable to previous estimates of the same size, though a slight underestimate (3%). Therefore, the methodology used to measure PMHS head mass is a reasonable approach compared to previous works.

For the small female PMHS, the recorded head mass was, on average, 2.9 kg. The PMHS mass is an underestimation (22%) compared to the head mass of the small female from Schneider et al., which was predicted as 3.7 kg. The values published by Schneider for the small female relied on the Young et al. sample from the 5^th^ to 95^th^ percentile females, with a mean height of 161 cm and weight of 64 kg. The Schneider prediction of head mass at the extremes of this region—for the 5^th^ percentile female—could have had a larger error due to sparser data compared to an estimate near the mean with denser data. This may, in part, explain the larger differences in observed PMHS head mass compared to previous estimates based on regressions using target anthropometry height and weight.

Regardless of why the measured small female PMHS head mass and previous estimates differ, the lighter head mass of the small female PMHS compared to anthropometrically matched surrogates (e.g., THOR-05F, GHBMC-F05) should be considered in the interpretation of the kinematics presented in this study. With heavier head forms, physical or computational surrogates would require a stiffer neck response to match PMHS data with a lighter head mass, which may exhibit smaller deformations due to the smaller inertial properties of the head.

## Applications of Data

These data were generated for refinement and validation of automobile occupant safety assessment tools such as HBMs and ATDs. For example, the PMHS testing described here was followed up by testing the THOR-05F and THOR-50 M dummies in matched conditions so that the biofidelity of their head-neck response can be evaluated [66]. This experimental dataset is also a critical piece of computational human body model assessments. For instance, in the GHBMC model, muscle activation can be superimposed on passive muscle responses. In this way, the GHBMC model can be assessed for how well it matches both a PMHS response (with no active muscle elements) and a volunteer response (with active muscle elements).

In parallel to direct comparison of the PMHS data to human body models for specific anthropometries, the PMHS data can be leveraged to assess scaling of responses from a midsize male surrogate to a small female surrogate. Scaling can be used when there is a paucity of data for a specific anthropometry, but concerns have been raised as to the fidelity of such estimates [67–69]. With these data, scaled estimates can be assessed for validity in neck biomechanics applications. It should be noted, however, that the assessment of scaling for PMHS only assesses differences based on geometry and passive stiffness; it does not assess how muscle contraction may vary between target anthropometries. Here, trajectories were qualitatively compared, as well as summary measures of motion (e.g., mean peak values). Tools like functional data analysis could provide a more informative and nuanced comparison across surrogate types and impact severities that assess the curves holistically.

Because this data was collected in conditions matched to original NBDL testing, it can be compared to the original male volunteer testing. Specifically, the midsize male PMHS testing can be compared to the midsize male volunteer testing to assess the effect of active musculature. Other NBDL configurations could also be explored in future, including lateral impacts and higher severity impacts to examine injury thresholds. The presented work highlights the importance of testing anthropometry-specific PMHS; differences in head kinematics between small female and midsize male, including larger magnitude downward motion, must be considered for surrogate development and refinement.

## Supplementary Information

Below is the link to the electronic supplementary material.

Supplementary file1 (DOCX 3954 KB)
