# Supplementary material for: Neck Motion and Injuries of Small Females and Midsize Males in Frontal Impacts at Two Severities
Source: Ann Biomed Eng. 2026 Jan 30;54(6):1895–913. doi: 10.1007/s10439-026-03981-6 (PMC13186882; doi:10.1007/s10439-026-03981-6)
Supplement: Supplementary file 1 — Supplementary file1 (DOCX 3954 KB) [file 10439_2026_3981_MOESM1_ESM.docx]

**Supplement**

Contents

[Appendix A: NBDL data used for sled input curves 1](#_Toc210406215)

[Appendix B: Positioning measures for PMHS 3](#_Toc210406216)

[Appendix C: NBDL data used for head and neck positioning guidance 5](#_Toc210406217)

[Appendix D. Head and T1 local coordinate systems. 8](#_Toc210406218)

[Appendix E: Individual curves and resulting corridors UVA PMHS kinematics 9](#_Toc210406219)

[Appendix F: Individual time-histories for UVA PMHS kinematics 13](#_Toc210406220)

[References 21](#_Toc210406221)

## Appendix A: NBDL data used for sled input curves

The sled pulses used for this test series were derived from original NBDL data, specifically the tests in **Table S1**. Some cases that were listed as nominally 14-g pulses were included in the 15-g group, as the nominally 14-g pulse had acquired acceleration curves that were within the range of variability of the acquired acceleration curves of the nominally 15-g pulses. These cases were selected because each subject was tested at least once at each severity of 3-g, 8-g, and 15-g. This article only examines the 3-g and 8-g sled pulses for the PMHS testing focused on kinematics.

For each acquired sled acceleration group, a characteristic average was calculated for use on the UVA SESA sled. Additionally, the characteristic averages for each the 3-g, 8-g, and 15-g sled acceleration profiles were modified for input into UVA SESA sled software by left-shifting by 35 ms (to eliminate the initial zero acceleration portion of the curve), inverting, and extrapolating the curves, as needed, to return to zero acceleration.

**Table S1**. NBDL reference tests used for generating target sled pulses.

| **NBDL Subject No.** | **NBDL Test Reference No.** | **NHTSA DB Test No.** | **Nominal Sled Acceleration Peak [g]** | **Acquired Sled Acceleration Peak [g]** |
| --- | --- | --- | --- | --- |
| 118 | 3796 | 1547 | 3 | 3 |
| 120 | 3793 | 1545 | 3 | 3 |
| 127 | 3794 | 1546 | 3 | 3 |
| 131 | 3804 | 1552 | 3 | 3 |
| 131 | 3840 | 1570 | 3 | 3 |
| 132 | 3805 | 1553 | 3 | 3 |
| 133 | 3841 | 1571 | 3 | 3 |
| 134 | 3807 | 1554 | 3 | 3 |
| 134 | 3842 | 1572 | 3 | 3 |
| 135 | 3808 | 1555 | 3 | 3 |
| 136 | 3809 | 1556 | 3 | 3 |
| 118 | 3886 | 1590 | 8 | 8 |
| 120 | 3882 | 1587 | 8 | 8 |
| 127 | 3883 | 1588 | 8 | 8 |
| 131 | 3894 | 1594 | 8 | 8 |
| 132 | 3997 | 1649 | 8 | 8 |
| 133 | 3895 | 1595 | 8 | 8 |
| 134 | 3890 | 1593 | 8 | 8 |
| 135 | 3898 | 1596 | 8 | 8 |
| 136 | 3901 | 1597 | 8 | 8 |
| 120 | 3954 | 1625 | 14 | 15 |
| 131 | 3987 | 1642 | 14 | 15 |
| 133 | 3963 | 1632 | 14 | 15 |
| 134 | 3968 | 1634 | 14 | 15 |
| 136 | 3962 | 1631 | 14 | 15 |
| 118 | 3958 | 1628 | 15 | 15 |
| 118 | 3969 | 1635 | 15 | 15 |
| 120 | 3972 | 1637 | 15 | 15 |
| 127 | 3959 | 1629 | 15 | 15 |
| 131 | 3990 | 1644 | 15 | 15 |
| 132 | 3957 | 1627 | 15 | 15 |
| 132 | 3982 | 1638 | 15 | 15 |
| 133 | 3986 | 1641 | 15 | 15 |
| 134 | 3983 | 1639 | 15 | 15 |
| 135 | 3965 | 1633 | 15 | 15 |
| 135 | 3970 | 1636 | 15 | 15 |

## Appendix B: Positioning measures for PMHS

The position of the PMHS was quantified by the angles described in Figure S2, Table S2, and Table S3. Values taken from reference dashed line; angles following convention shown with solid line are positive.


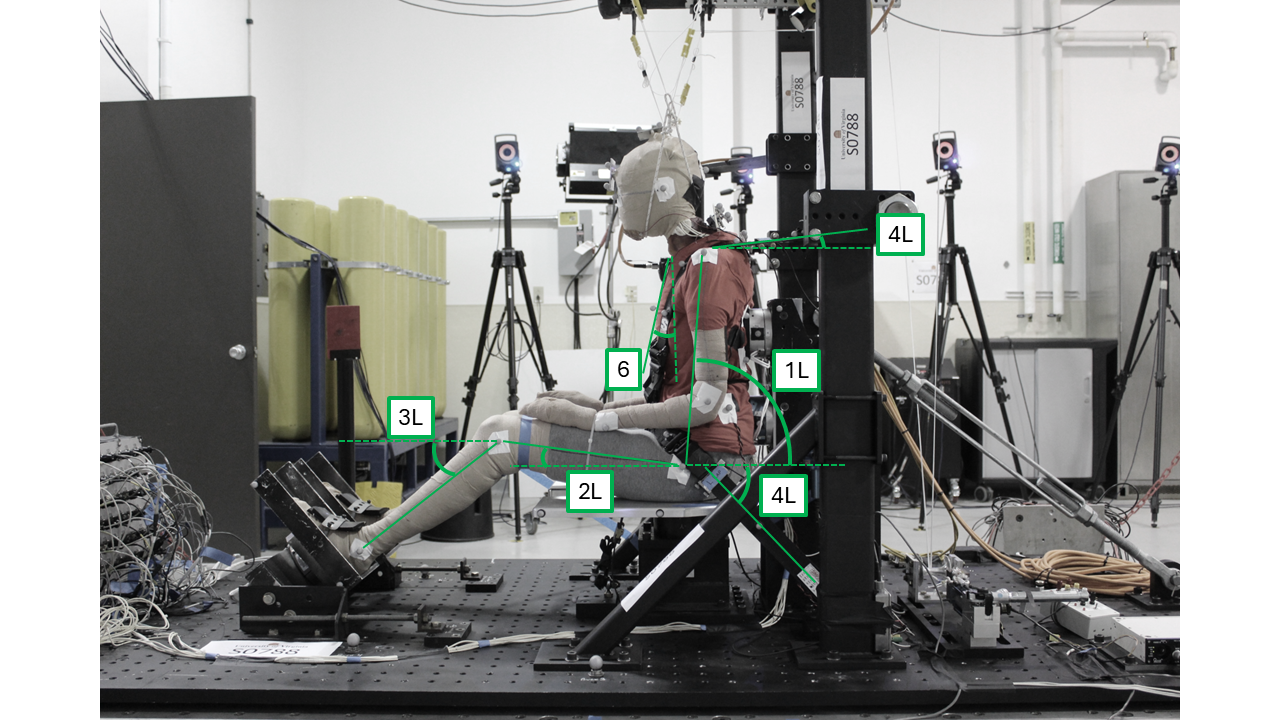


**Figure S2.** A left sagittal view with measurements to quantify the position of the PMHS.

**Table S2.** Definition of measurements in Figure S2.

| **Measure** | **Description** |
| --- | --- |
| 1R | Right side torso angle (H-pt (femoral) to shoulder (humeral) pt) |
| 1L | Left side torso angle (H-pt (femoral) to shoulder (humeral) pt) |
| 2R | Right side thigh (femur) angle |
| 2L | Left side thigh (femur) angle |
| 3R | Right side leg (fibula) angle |
| 3L | Left side leg (fibula) angle |
| 4R | Right side shoulder belt take-off angle, sagittal plane |
| 4L | Left side shoulder belt take-off angle, sagittal plane |
| 5R | Right side lap belt take-off angle, sagittal plane |
| 5L | Left side lap belt take-off angle, sagittal plane |
| 6 | Sternum angle |

**Table S3.** Recorded values for small female PMHS position.

| PMHS ID | Test | 1R | 1L | 2R | 2L | 3R | 3L | 4R | 4L | 5R | 5L | 6 |
| --- | --- | --- | --- | --- | --- | --- | --- | --- | --- | --- | --- | --- |
| 1046F | S0785 | 84 | 81 | 11 | 13 | 36 | 36 | 2 | 2 | 44 | 45 | 84 |
| 1046F | S0786 | 83 | 78 | 11 | 15 | 37 | 36 | 4 | 4 | 44 | 46 | 84 |
| 1042F | S0787 | 86 | 84 | 8 | 8 | 40 | 40 | 3 | 2 | 47 | 45 | 80 |
| 1042F | S0788 | 83 | 84 | 8 | 7 | 40 | 40 | 3 | 3 | 47 | 46 | 77 |
| 1048F | S0789 | 81 | 81 | 8 | 7 | 32 | 31 | 1 | 3 | 43 | 44 | 89 |
| 1048F | S0790 | 80 | 79 | 8 | 8 | 30 | 30 | 1 | 2 | 43 | 42 | 88 |
| 1051F | S0797 | 82 | 82 | 10 | 10 | 35 | 34 | 0 | 0 | 43 | 43 | 70 |
| 1051F | S0798 | 81 | 80 | 10 | 10 | 35 | 35 | 1 | 1 | 42 | 43 | 65 |
| 1054F | S0799 | 85 | 85 | 9 | 8 | 37 | 36 | 5 | 5 | 41 | 43 | 87 |
| 1054F | S0800 | 85 | 86 | 9 | 8 | 37 | 36 | 6 | 5 | 41 | 42 | 88 |
| 1049F | S0801 | 86 | 83 | 7 | 8 | 39 | 41 | -3 | 2.5 | 47 | 46 | 85 |
| 1049F | S0802 | 86 | 80 | 7 | 9 | 38 | 41 | 4.5 | 4 | 47 | 47 | 82 |
| Avg | | 84 | 82 | 9 | 9 | 36 | 36 | 3 | 3 | 44 | 44 | 82 |
| StD | | 2 | 3 | 1 | 2 | 3 | 4 | 2 | 2 | 2 | 2 | 8 |
| Min | | 81 | 79 | 7 | 7 | 33 | 33 | 1 | 1 | 42 | 43 | 74 |
| Max | | 86 | 84 | 10 | 12 | 39 | 40 | 5 | 4 | 46 | 46 | 89 |

**Table S4.** Recorded values for midsize male PMHS position.

| PMHS ID | Test | 1R | 1L | 2R | 2L | 3R | 3L | 4R | 4L | 5R | 5L | 6 |
| --- | --- | --- | --- | --- | --- | --- | --- | --- | --- | --- | --- | --- |
| 1032M | S0791 | 84 | 84 | 13 | 11 | 38 | 37 | 3 | 4 | 44 | 45 | 75 |
| 1032M | S0792 | 83 | 81 | 14 | 12 | 37 | 37 | 4 | 5 | 45 | 44 | 77 |
| 1038M | S0793 | 85 | 86 | 10 | 10 | 37 | 38 | 2 | 1 | 44 | 45 | 84 |
| 1038M | S0794 | 88 | 87 | 9 | 11 | 37 | 39 | 3 | 2 | 44 | 45 | 85 |
| 1031M | S0795 | 86 | 87 | 10 | 10 | 33 | 36 | 2 | 1 | 44 | 44 | 75 |
| 1031M | S0796 | 85 | 85 | 10 | 9 | 33 | 34 | 2 | 2 | 43 | 43 | 120 |
| Avg | | 85 | 85 | 11 | 11 | 36 | 37 | 3 | 3 | 44 | 44 | 86 |
| StD | | 2 | 2 | 2 | 1 | 2 | 2 | 1 | 2 | 1 | 1 | 17 |
| Min | | 83 | 83 | 9 | 9 | 34 | 35 | 2 | 1 | 43 | 44 | 69 |
| Max | | 87 | 87 | 13 | 12 | 38 | 39 | 3 | 4 | 45 | 45 | 103 |

## Appendix C: NBDL data used for head and neck positioning guidance

The in-position measurements for the NBDL subjects listed in **Table S1** was not available in the NHTSA Biomechanics Database. However, available literature on NBDL describes positioning for similar test runs. An NBDL sensitivity study regarding head and neck position on earlier test runs under 6-g and 10-g pulses explored four possible positions: “neck up, chin up” (“NUCU”), “neck up, chin down” (“NUCD”), “neck forward, chin up” (“NFCU”), and “neck forward, chin down” (“NFCD”) (**Figure S3**) [1]. Quantitative measures for these positions were described by the head and neck angles in **Figure S4**. For the test runs in **Table S1**, which are the NBDL tests used to derive the sled pulses in this study, the subjects were positioned in the “NUCU” position [2]. The head and neck angles for subjects in the Ewing et al.1975 positioning sensitivity study in the “NUCU” position are in **Table S2**. For the UVA PMHS tests, the average head and neck angles from **Table S2** (6-g and 10-g) were used for positioning guidance based on external landmarks after being converted to the J211 sign convention (i.e., flipping the sign).


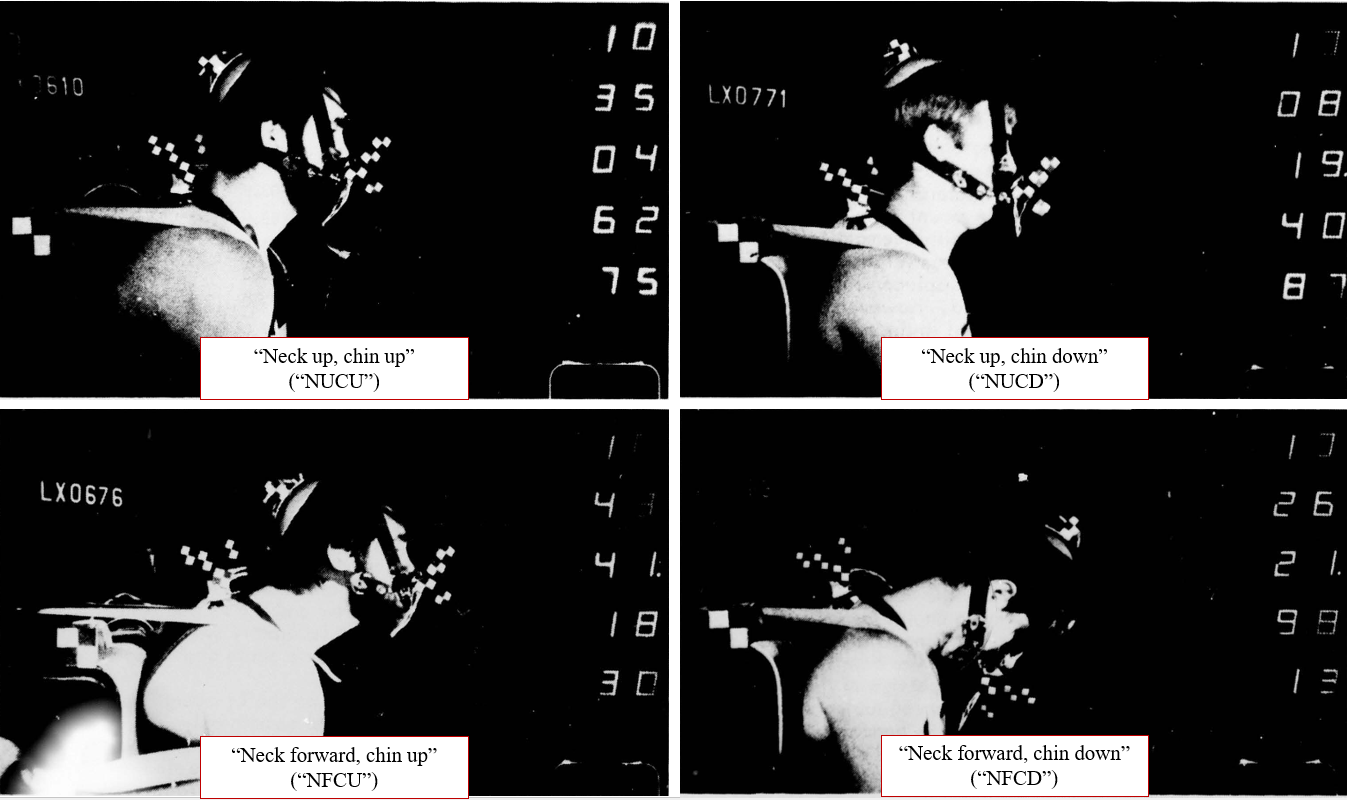


**Figure S3.** Variations of head and neck position explored in earlier NBDL study [1].


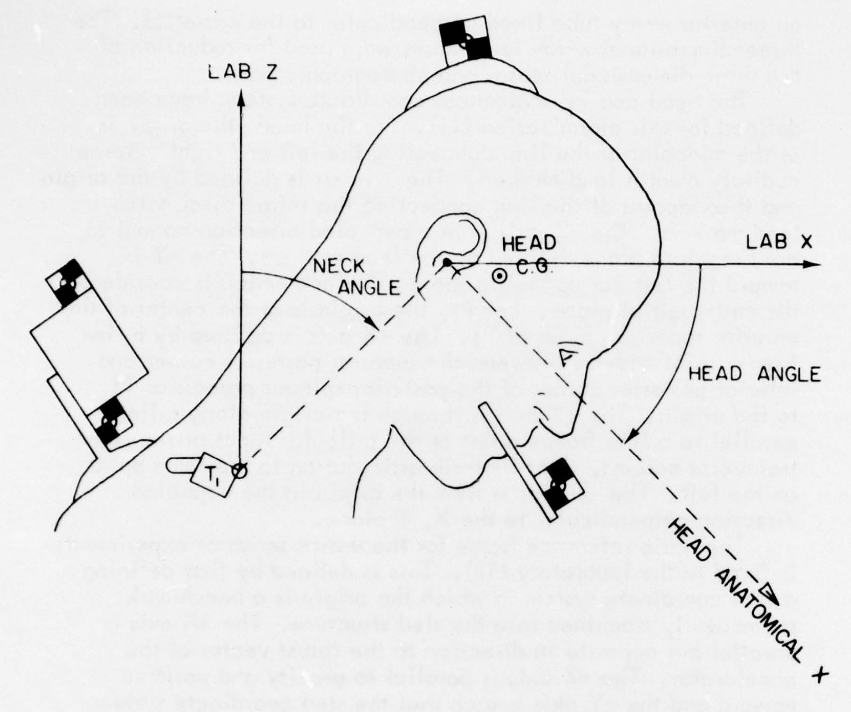


**Figure S4.** Definitions of head and neck angles by NBDL researchers [1].

**Table S2.** Reference values for target NBDL head and neck angles [1]. Note these are in the original sign convention of **Figure S4**, not J211.

|  | **Head angle [deg]** | | **Neck angle [deg]** | |
| --- | --- | --- | --- | --- |
| **NBDL Subject No.** | **6-g** | **10-g** | **6-g** | **10-g** |
| 29 | 1.3 | -4.5 | 20.6 | 27.6 |
| 32 | -10.5 | -16.3 | 20.7 | 11.3 |
| 33 | 1.9 | 5.6 | 23.9 | 20.4 |
| 34 | -9.3 | -13.5 | 18.8 | 10.2 |
| 35 | 9.4 | 8.2 | 18.8 | 19.6 |
| 37 | -2.9 | -5.9 | 25.1 | 21.2 |
| 38 | 6.1 | 4.5 | 19.0 | 20.9 |
| 39 | -16.1 | -20.4 | 12.9 | 5.5 |
| 41 | -2.9 | 8.6 | 18.0 | 21.1 |
| 42 | 2.4 | 6.9 | 20.1 | 26.8 |
| 43 | -1.2 | -0.4 | 19.0 | 28.8 |
| 44 | 5.0 | 10.7 | 24.6 | 32.6 |
| 45 | -0.1 | 10.0 | 22.6 | 30.8 |
| Mean | -1.3 | -0.5 | 20.3 | 21.3 |
| **Mean 6-g and 10-g** | **-0.9** | | **20.8** | |
| St. dev. | 7.1 | 10.7 | 3.3 | 8.3 |
| Max | 9.4 | 10.7 | 25.1 | 32.6 |
| Min | -16.1 | -20.4 | 12.9 | 5.5 |

## Appendix D. Head and T1 local coordinate systems.

Local coordinate systems were defined on the head and T1 [3] (**Figure S4**). The origin and reference point of the head was at the midpoint of the left and right zygomatic processes. The Y-axis of the head passed from left to right zygomatic process, positive to the right. The Z-axis of the head was perpendicular to the plane containing the Y-axis and the infraorbitales, positive inferiorly. The X-axis of the head was perpendicular to the Y- and Z-axes, positive anteriorly. The origin and reference point of T1 was the center of the vertebral body. The Z-axis of T1 passed through the centers of the superior and inferior endplates, positive inferiorly. The X-axis of T1 was perpendicular to the Z-axis and the line passing through the points of maximum concavity in the superior aspects of the left and right pedicles, positive anteriorly. The Y-axis of T1 was perpendicular to the Z- and X-axes, positive to the right.

**Figure S4**. Head (left) and T1 (right) local coordinate systems.

## Appendix E: Individual curves and resulting corridors UVA PMHS kinematics

**Figures S5** to **S8** show individual PMHS subject curves and resulting corridors for head and T1 motion.


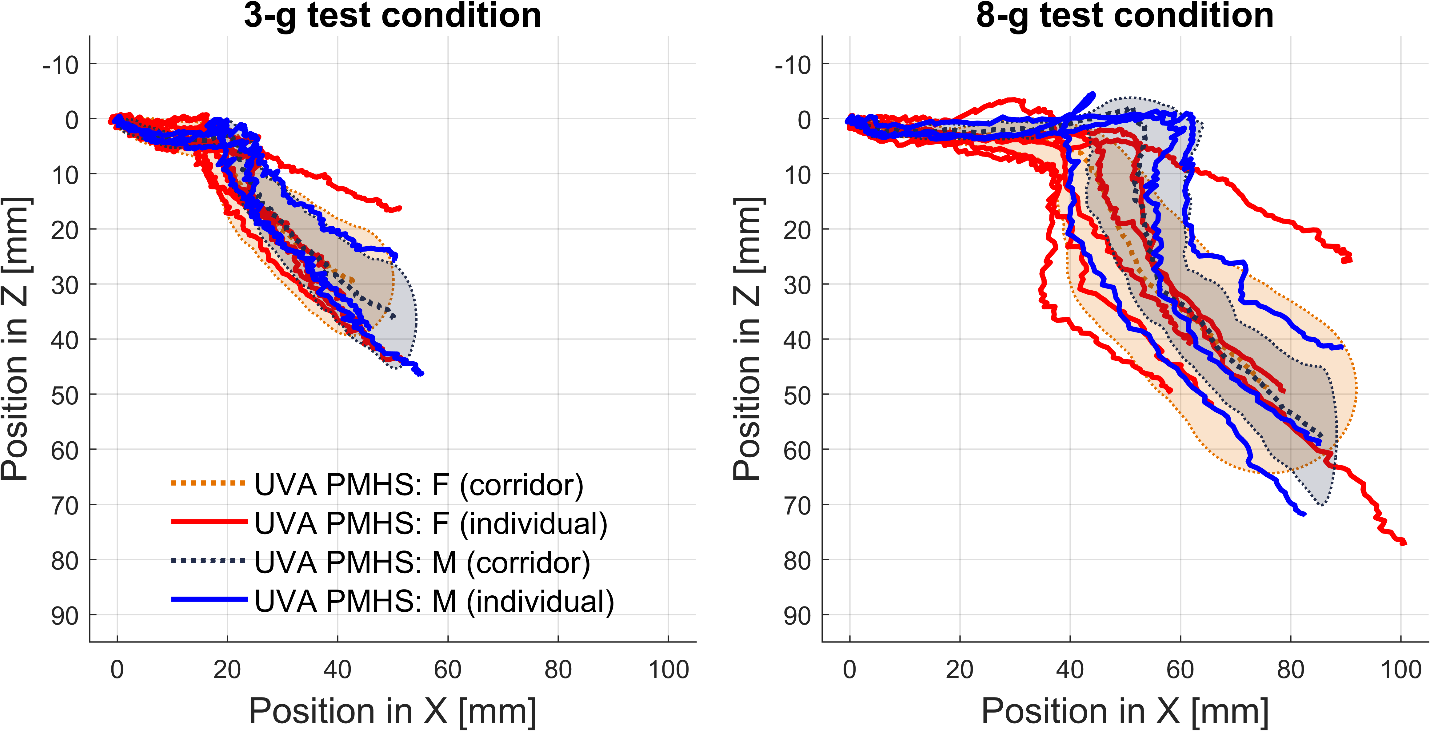


**Figure S5.** T1 sagittal displacement for UVA PMHS. Data is truncated based on maximum head Z displacement to exclude head rebound. Individual subject curves and corridors (characteristic average ± 1 standard deviation) are shown.


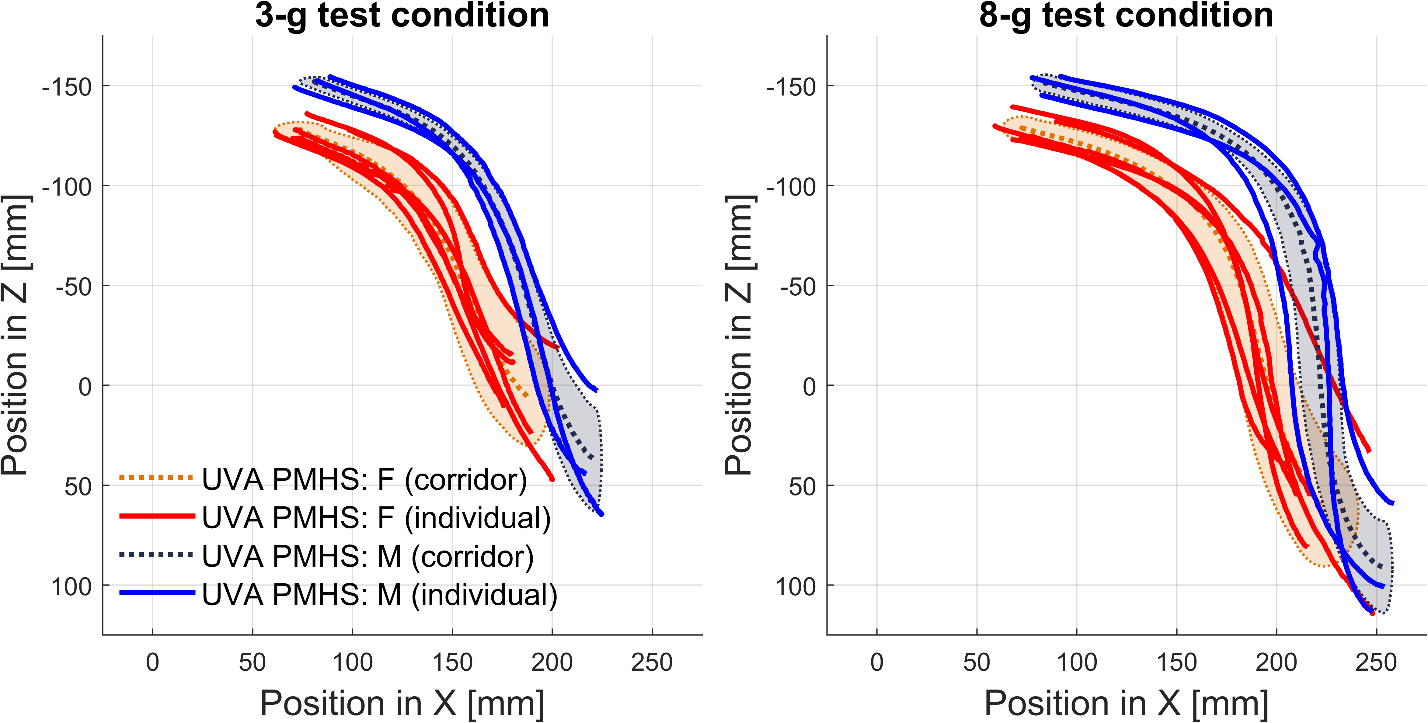


**Figure S6.** Head sagittal position for UVA PMHS. Data is truncated based on maximum head Z displacement to exclude head rebound. Individual subject curves and corridors (characteristic average ± 1 standard deviation) are shown.


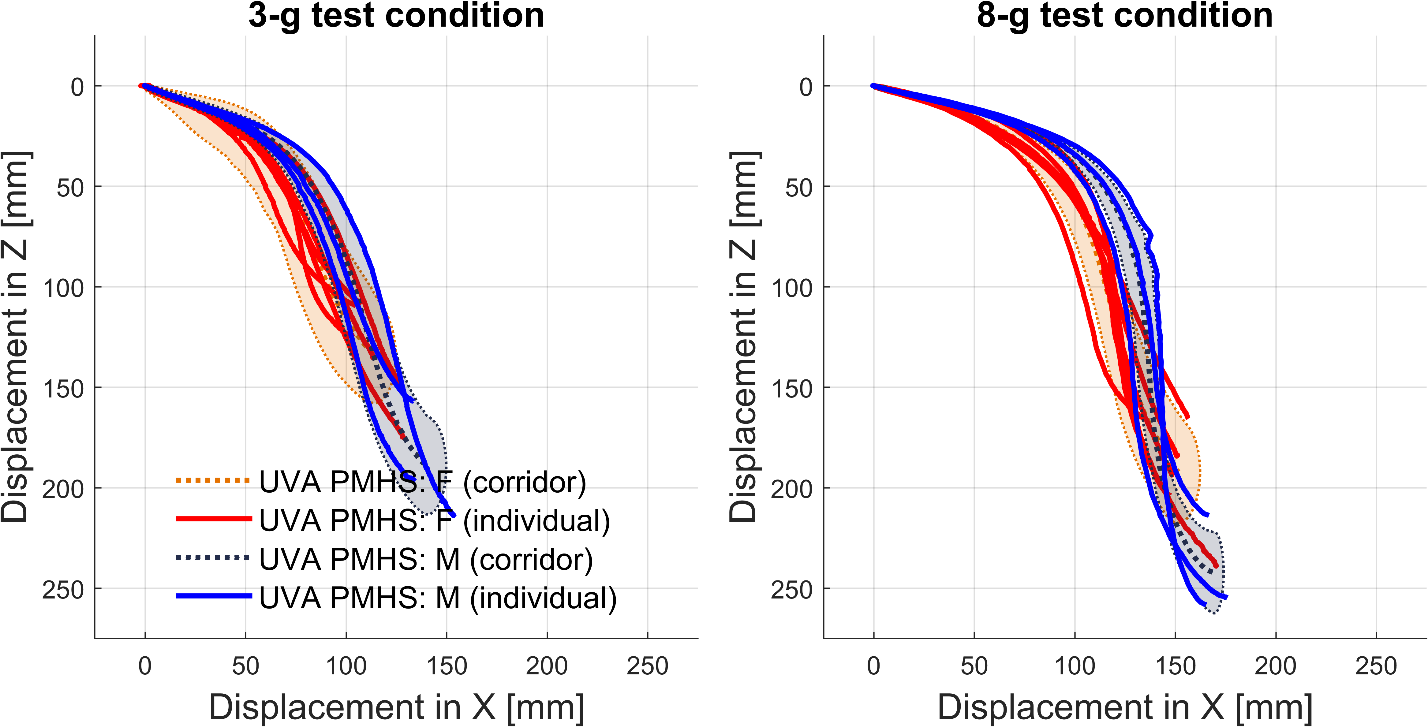


**Figure S7.** Head sagittal displacement for UVA PMHS. Data is truncated based on maximum head Z displacement to exclude head rebound. Individual subject curves and corridors (characteristic average ± 1 standard deviation) are shown.


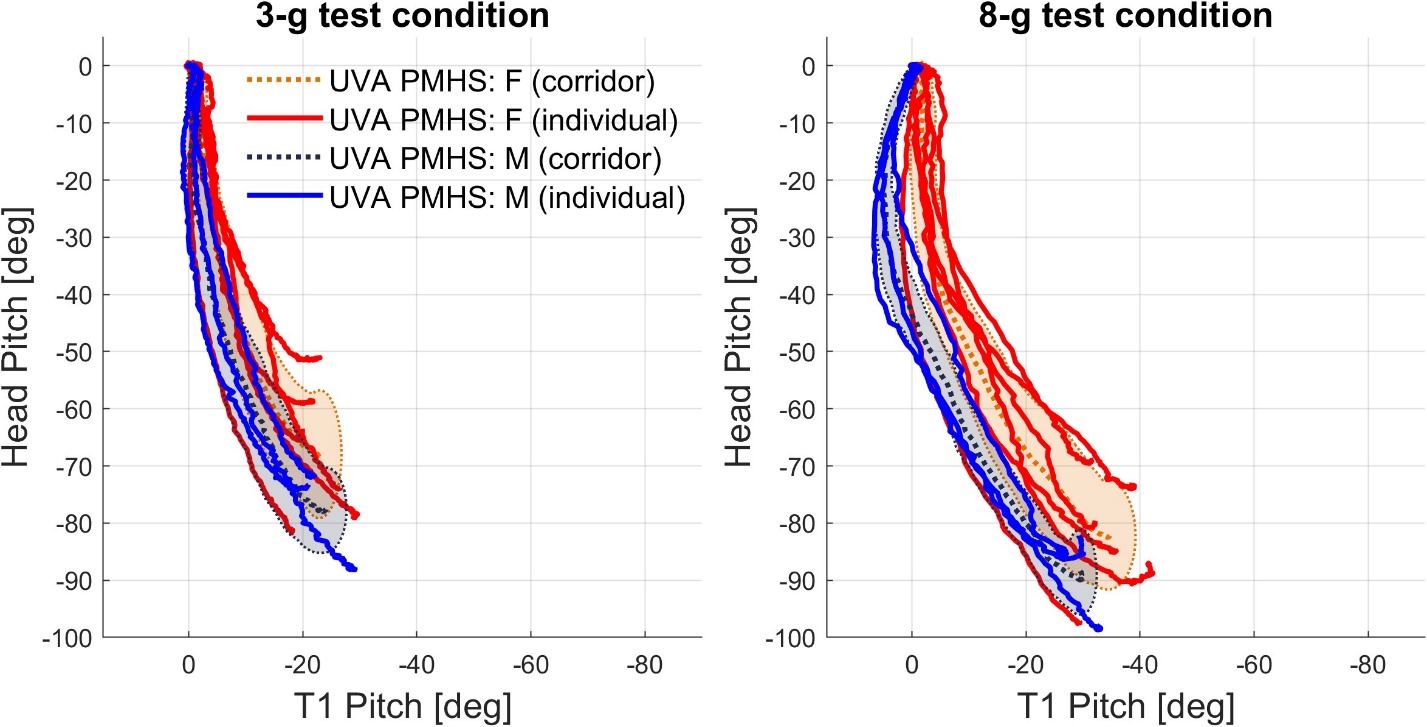


**Figure S8.** Head pitch relative to T1 pitch for UVA PMHS. Data is truncated based on maximum head Z displacement to exclude head rebound. Individual subject curves and corridors (characteristic average ± 1 standard deviation) are shown.

## Appendix F: Individual time-histories for UVA PMHS kinematics

**Figures S9** to **S14** show kinematic time histories of individual PMHS subjects for head and T1 motion. Note that these time-based histories were not truncated based on maximum Z excursion like the corridors in the main text were. The 3-g tests include data for 350 ms; the 8-g tests include data for 250 ms. The difference in time duration in analyses is because more data could be collected for the 3-g impact, as the PMHS remained in the Vicon capture volume for longer.


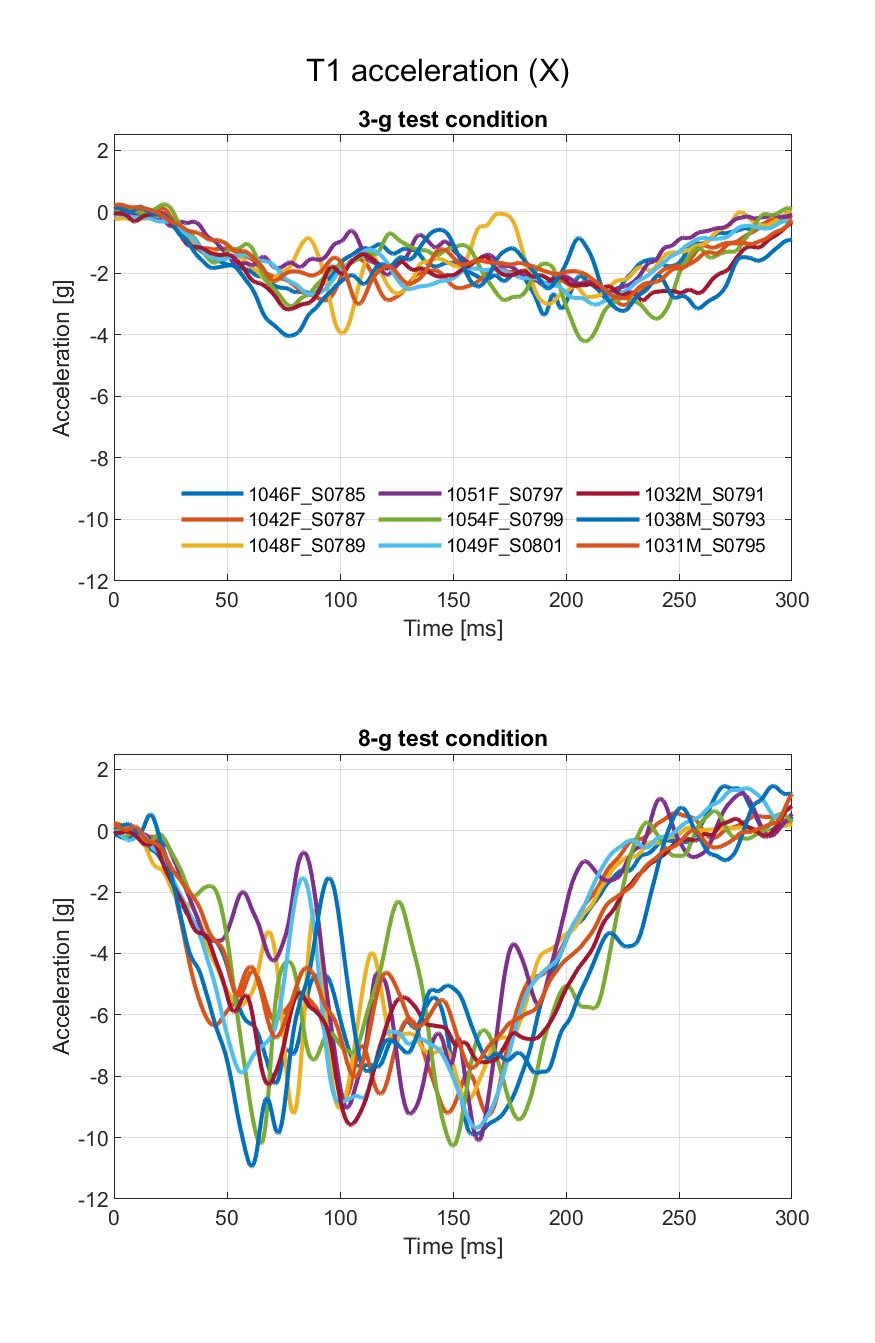


**Figure S9.** T1 accelerations in X for UVA PMHS.


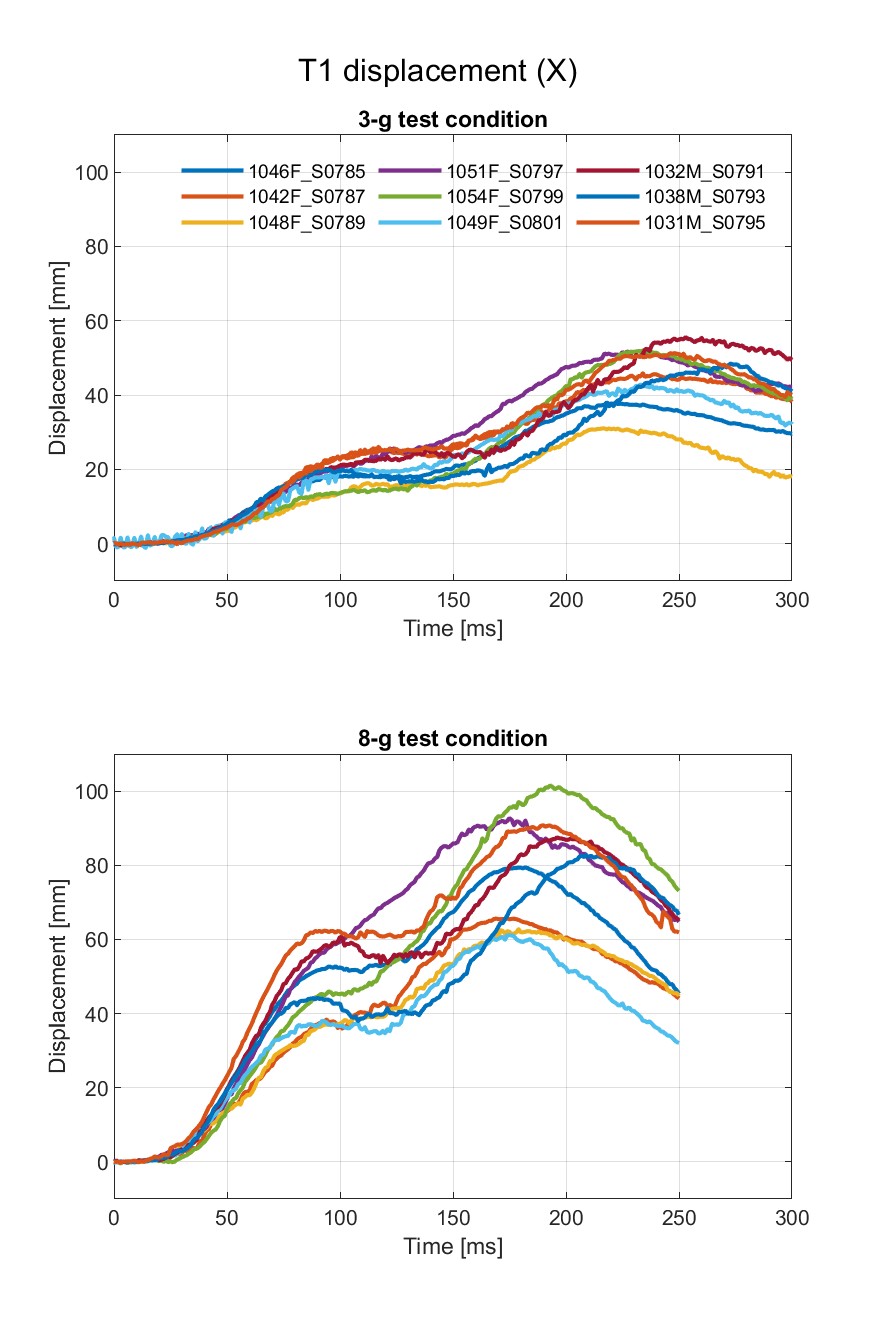


**Figure S10.** T1 displacement in X for UVA PMHS.


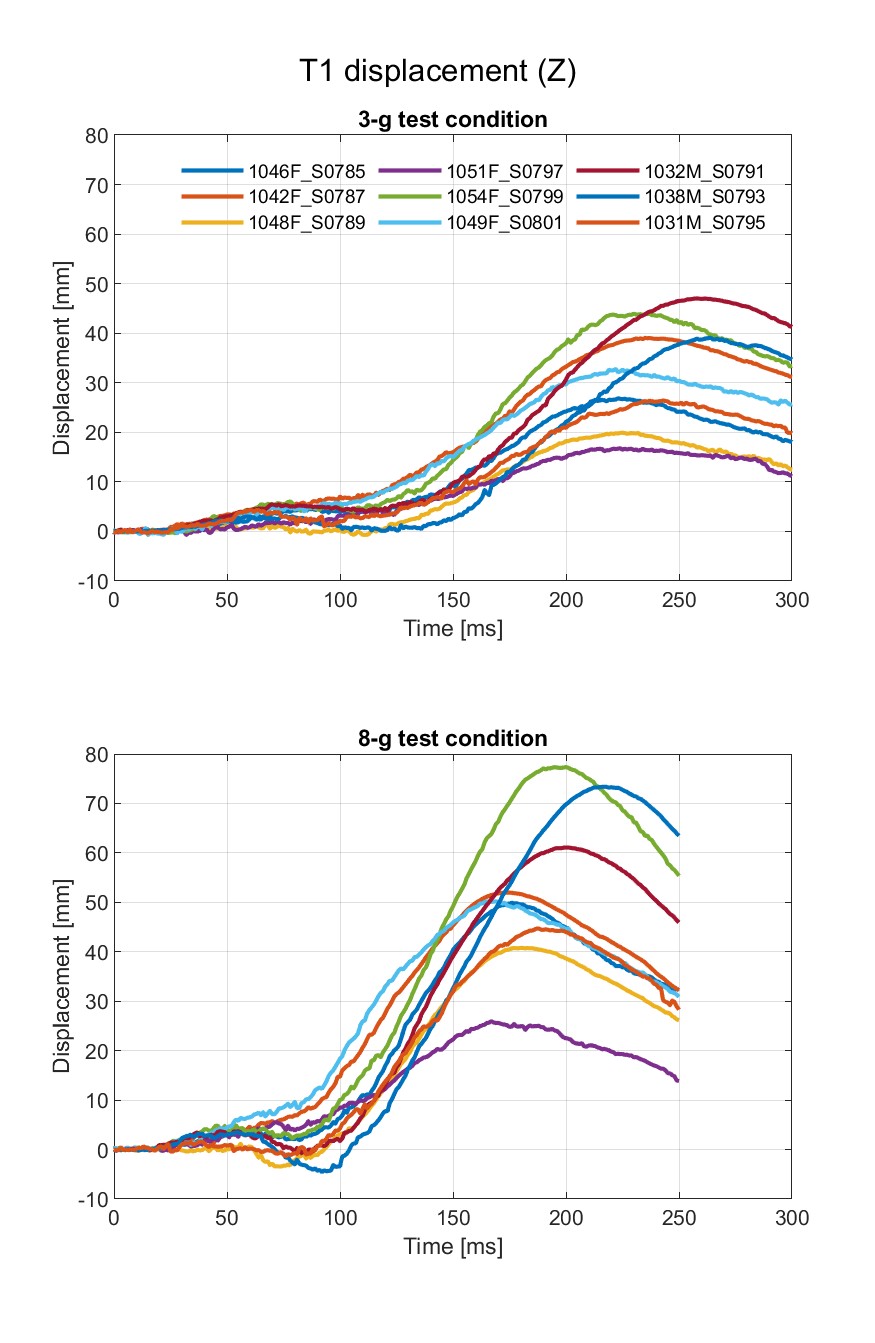


**Figure S11.** T1 displacement in Z for UVA PMHS.


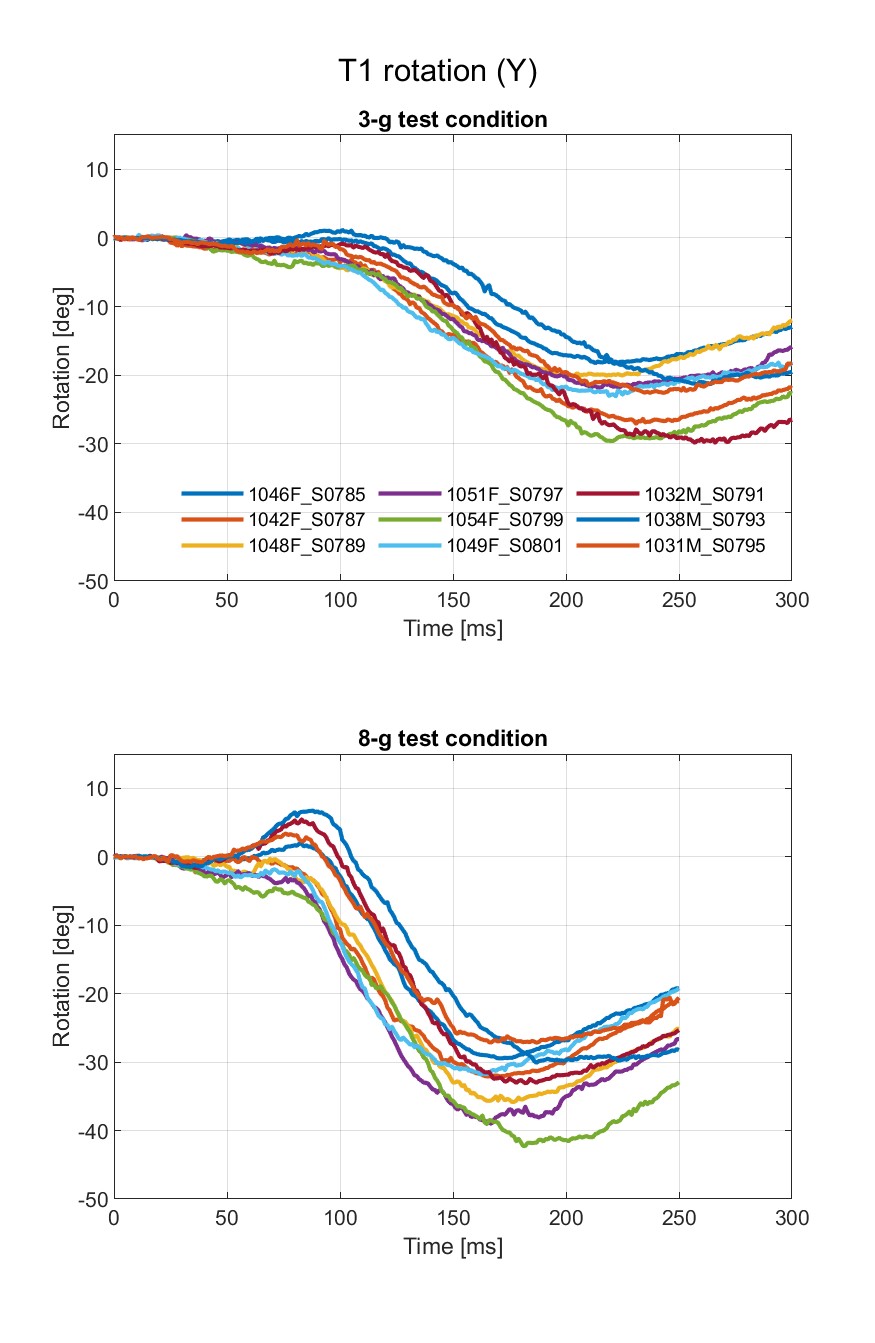


**Figure S12.** T1 rotation about Y for UVA PMHS.


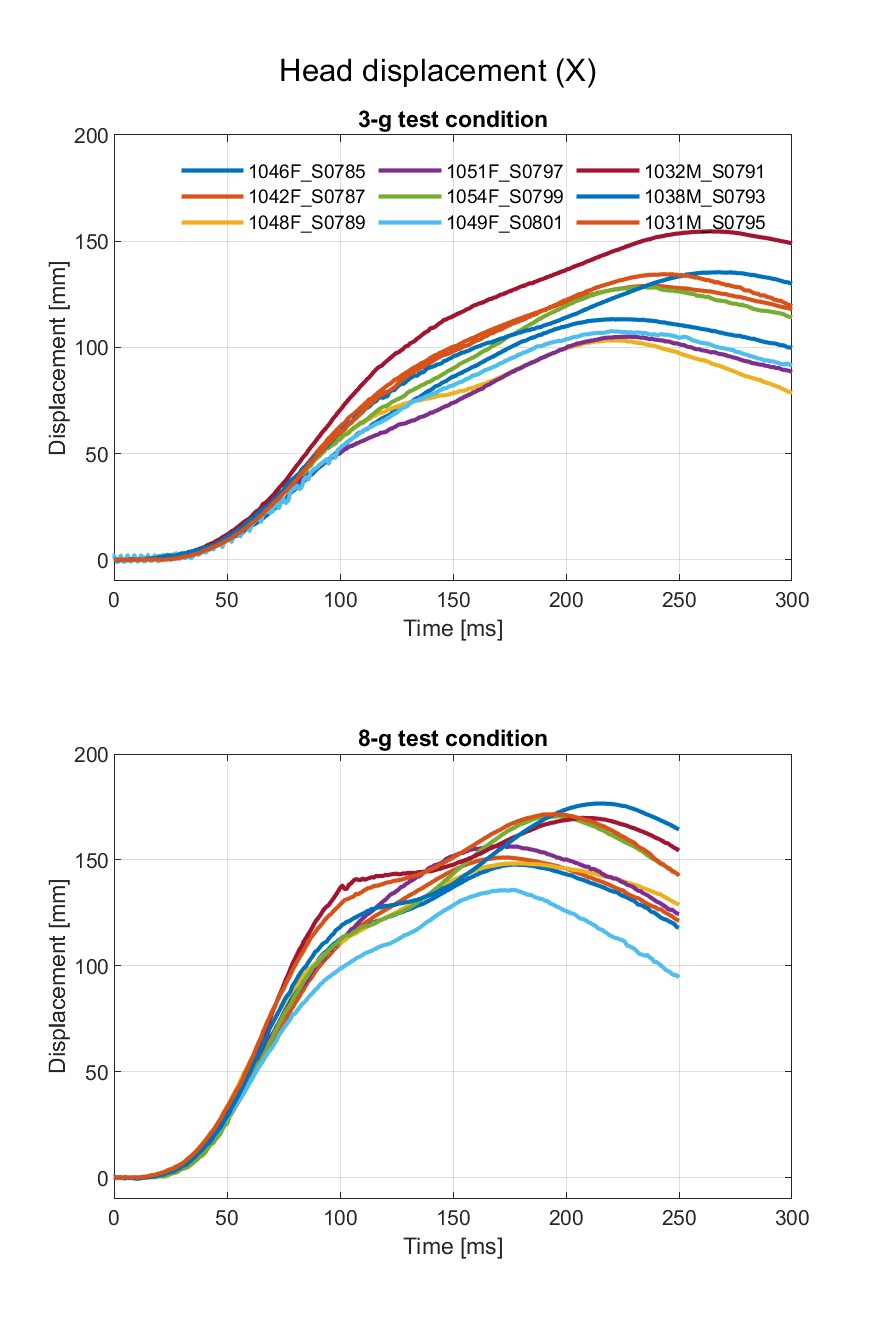


**Figure S13.** Head displacement in X for UVA PMHS.


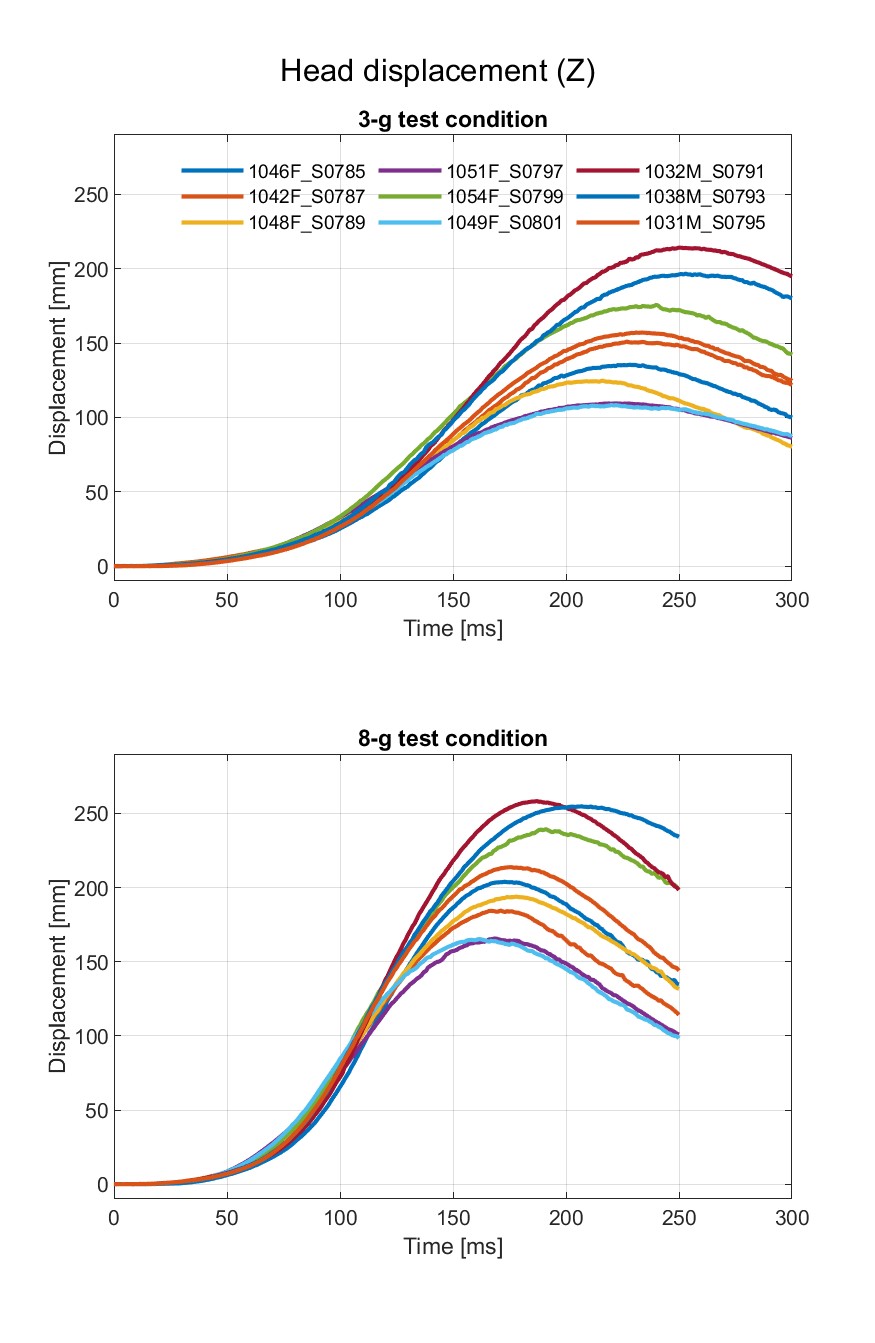


**Figure S14.** Head displacement in Z for UVA PMHS.


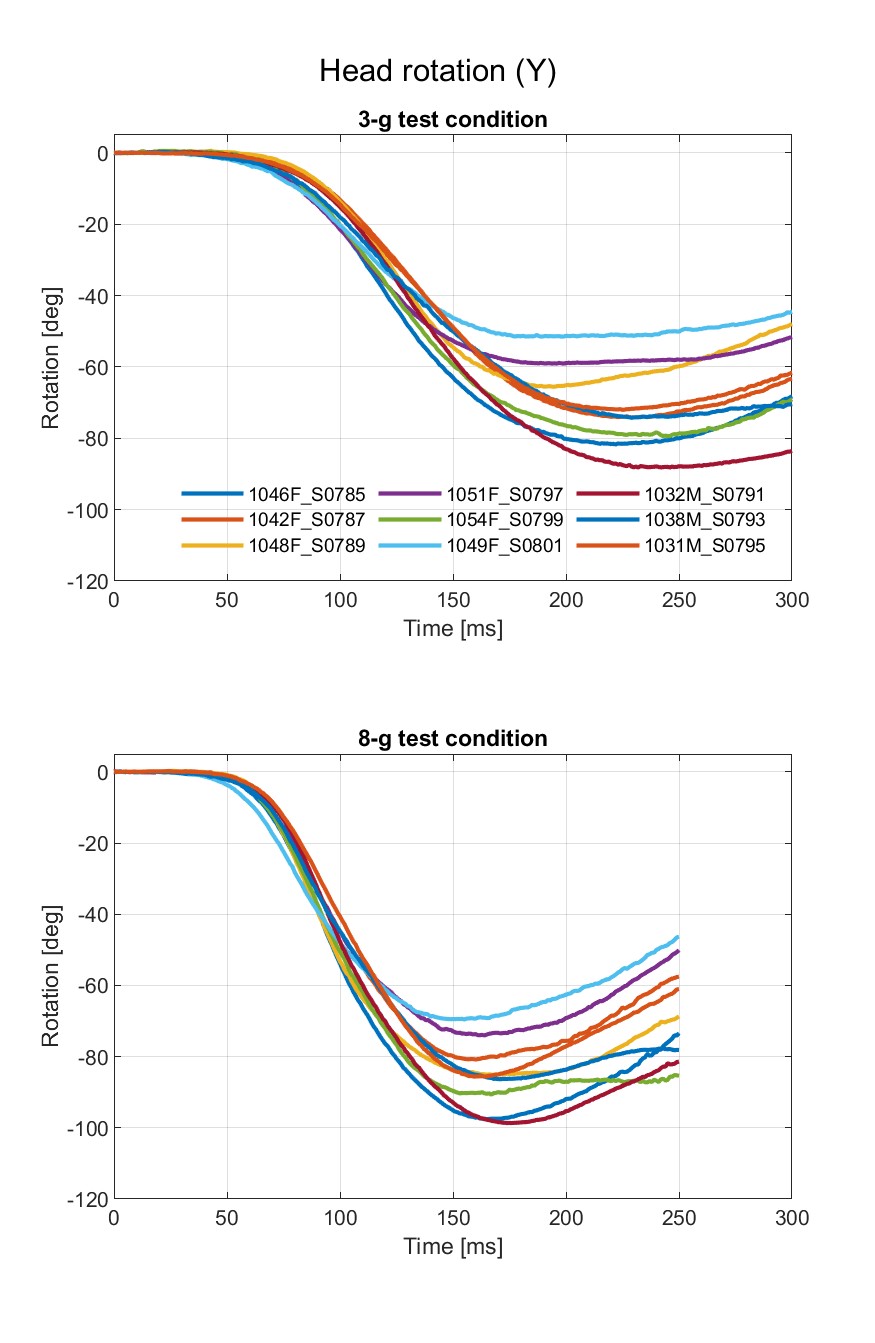


**Figure S15.** Head rotation about Y for UVA PMHS.

## References

[1] C. L. Ewing, D. J. Thomas, L. Lustick, G. Becker, G. C. Willems, and W. H. Muzzy, “The Effect of the Initial Position of the Head and Neck on the Dynamic Response of the Human Head and Neck to -Gx Impact Acceleration,” Society of Automotive Engineers, Warrendale, PA, Proceedings, 1975. Accessed: Nov. 07, 2022. [Online]. Available: https://apps.dtic.mil/sti/citations/ADA043031

[2] C. H. Spenny and John A. Volpe National Transportation Systems Center (U.S.), “Analysis of Head Response to Torso Acceleration. Vol. I - Development of Performance Requirements,” DOT-TSC-NHTSA-85-5, Nov. 1987. Accessed: Nov. 08, 2022. [Online]. Available: https://rosap.ntl.bts.gov/view/dot/11956

[3] G. Shaw *et al.*, “Impact Response of Restrained PMHS in Frontal Sled Tests: Skeletal Deformation Patterns Under Seat Belt Loading,” SAE International, Warrendale, PA, SAE Technical Paper 2009-22–0001, Nov. 2009. doi: 10.4271/2009-22-0001.
